# Long non-coding RNA LPP-AS2 promotes glioma tumorigenesis via miR-7-5p/EGFR/PI3K/AKT/c-MYC feedback loop

**DOI:** 10.1186/s13046-020-01695-8

**Published:** 2020-09-22

**Authors:** Xiaoming Zhang, Wanxiang Niu, Maolin Mu, Shanshan Hu, Chaoshi Niu

**Affiliations:** 1 Department of Neurosurgery, The First Affiliated Hospital of USTC, Division of Life Sciences and Medicine, University of Science and Technology of China, Hefei, Anhui 230001 P.R. China; 2 Anhui Key Laboratory of Brain Function and Diseases, Hefei, Anhui 230001 P.R. China; 3 Anhui Provincial Stereotactic Neurosurgical Institute, Hefei, Anhui 230001 P.R. China

**Keywords:** lncRNA, LPP-AS2, EGFR, miR-7-5p, Glioma, PI3K/AKT/c-MYC pathway

## Abstract

**Background:**

Glioblastoma is the most common primary malignant intracranial tumor with poor clinical prognosis in adults. Accumulating evidence indicates that long non-coding RNAs (lncRNAs) function as important regulators in cancer progression, including glioblastoma. Here, we identified a new lncRNA LPP antisense RNA-2 (LPP-AS2) and investigated its function and mechanism in the development of glioma.

**Methods:**

High-throughput RNA sequencing was performed to discriminate differentially expressed lncRNAs and mRNAs between glioma tissues and normal brain tissues. Expression of LPP-AS2, epidermal growth factor receptor (EGFR) and miR-7-5p in glioma tissues and cell lines was detected by real-time quantitative PCR (RT-qPCR), and the functions of lncRNA LPP-AS2 in glioma were assessed by in vivo and in vitro assays. Insight into the underlying mechanism of competitive endogenous RNAs (ceRNAs) was obtained via bioinformatic analysis, dual luciferase reporter assays, RNA pulldown assays, RNA immunoprecipitation (RIP) and rescue experiments.

**Results:**

The results of high-throughput RNA-seq indicated lncRNA LPP-AS2 was upregulated in glioma tissues and further confirmed by RT-qPCR. Higher LPP-AS2 expression was related to a poor prognosis in glioma patients. Based on functional studies, LPP-AS2 depletion inhibited glioma cell proliferation, invasion and promoted apoptosis in vitro and restrained tumor growth in vivo, overexpression of LPP-AS2 resulted in the opposite effects. In addition, LPP-AS2 and EGFR were observed in co-expression networks. LPP-AS2 was found to function as a ceRNA to regulate EGFR expression by sponging miR-7-5p in glioma cells. The result of chromatin immunoprecipitation (ChIP) assays validated that c-MYC binds directly to the promoter region of LPP-AS2. As a downstream protein of EGFR, c-MYC was modulated by LPP-AS2 and in turn enhanced LPP-AS2 expression. Thus, lncRNA LPP-AS2 promoted glioma tumorigenesis via a miR-7-5p/EGFR/PI3K/AKT/c-MYC feedback loop.

**Conclusions:**

Our study elucidated that LPP-AS2 acted as an oncogene through a novel molecular pathway in glioma and might be a potential therapeutic approach for glioma diagnosis, therapy and prognosis.

## Background

As the most common primary and devastating intracranial neoplasm, glioblastoma is characterized by a high incidence and an extremely poor prognosis and is especially difficult to diagnose early [1]. Gliomas are classified into numerical grades (I–IV) according to the pathological classification of the World Health Organization (WHO) [2]. At present, despite undergoing aggressive surgical resection and a regular course of postoperative radiotherapy or chemotherapy, the overall 5-year survival rate for glioblastoma patients remains poor [3, 4]. A number of crucial factors that contribute to the resistance of glioblastoma to standard therapeutic therapy have been reported, including genetic heterogeneity, multiple genetic lesions and dysregulated pathways [5, 6]. Nonetheless, more in-depth research and a better understanding of the molecular mechanisms underlying gliomagenesis that contribute to the diagnosis and treatment of glioma are urgently needed.

Non-coding RNAs are a class of RNAs without protein-coding potential that are produced by transcription of most genes [7]; they are divided into short non-coding RNAs and long non-coding RNAs (lncRNAs) based on their lengths [8]. LncRNAs are a major class of ncRNAs greater than 200 nucleotides in size; these molecules are mostly produced by RNA polymerase II transcription and possess 5′-cap and 3′-poly(A) structures [9, 10]. Increasing numbers of studies indicate that lncRNAs are not only involved in the regulation of various important biological processes, including chromatin modification, alternative splicing, epigenetic regulation, scaffolds and decoys with other molecules, transcriptional and post-transcriptional regulation [11–15], but also exert crucial functions in cell development and disease [16–18]. For instance, lncRNAs, such as MALAT-1, HOTAIR, PVT1 and NEAT1 may be of great significance in carcinogenesis [19–23]. In addition, the lncRNA MIR155HG is upregulated in glioma and inhibits mesenchymal transition by directly targeting miR-155 [24]. However, the clinical significance and underlying mechanism of lncRNAs, especially in glioma, need to be further explored.

LPP-AS2 (NR_036497.1) is a recently detected lncRNA with a length of 2897 bp. LPP-AS2 was considered to be associated with recurrent soft tissue sarcoma and breast cancer, as based on bioinformatic analysis without experimental verification [25, 26]. Nevertheless, MYC-repressed lncRNA LPP-AS2 was found to be downregulated in colorectal cancer and inhibited cell proliferation by regulating GADD45A [27]. However, the molecular mechanism and clinical prognostic value of LPP-AS2 in glioma have not been elucidated.

MicroRNAs (miRNAs) are endogenous ~ 23 nucleotide RNAs that are processed from stem-loop regions of longer RNA transcripts, and regulate gene expression by base-pairing to mRNAs to induce mRNA decay and inhibit translation [28–30]. In recent years, hundreds of different miRNAs have been identified in humans, many of which play increasingly recognized roles in the development of disease including cancer [31, 32]. For instance, miR-7-5p was downregulated and exerted tumor suppressor functions in different cancers [33–35]. Importantly, the suppressive effect of miR-7-5p in glioma has not been fully clarified.

EGFR is a tyrosine kinase receptor that plays an integral role in signaling pathways that control normal and aberrant cell growth [36]. Recent studies suggested that EGFR was dysregulated in many different tumor types, including melanoma [37], breast cancer [38], non-small cell lung cancer [39] and colorectal cancer [40]. Moreover, EGFR was important for glioblastoma cell growth and tumor initiation [41, 42], though the potential upstream regulatory mechanism of EGFR in glioblastoma remains unknown.

In the present study, we applied next-generation analysis and identified a lncRNA LPP-AS2, that is upregulated in glioblastoma; of note, the biological implications of LPP-AS2 in tumorigenesis have not been described to date. Higher LPP-AS2 expression was associated with poorer prognosis in glioblastoma patients. Subsequent in vitro and in vivo functional assays showed that LPP-AS2 affected glioma cell proliferation, apoptosis and invasion. Mechanistically, we found that LPP-AS2 sponged miR-7-5p to increase EGFR expression and activated the PI3K/AKT/c-MYC pathway. Moreover, LPP-AS2 was directly and transcriptionally regulated by the c-MYC protein and inversely increased c-MYC protein levels. Our study revealed that LPP-AS2 exerted as an oncogene in a positive feedback loop and might be a novel therapeutic target for the treatment of glioblastomas.

## Materials and methods

### Patients and clinical samples

In the study, the cohort of tissue specimens contained 106 glioma tissues and 23 normal brain tissues obtained from the Department of Neurosurgery of the First Affiliated Hospital of USTC between February 2014 and September 2019. All tumor samples were clinicopathologically confirmed as glioma (12 Grade I, 15 Grade II, 30 Grade III and 49 Grade IV). All of glioma tissues were astrocytoma and IDH wildtype. Normal brain tissues were collected from patients undergoing brain tissue resection due to craniocerebral injury. All samples were immediately stored in liquid nitrogen with RNAhold (TransGen). Written informed consent was obtained from all patients, and the study was approved by the Ethics Committee of the First Affiliated Hospital of USTC (2016-ky072). The basic characteristics of the included patients are shown in Table 1.

**Table 1:** Basic information of glioma patients and craniocerebral trauma patients

Clinical pathological parameters of patients		
Type	glioma	normal
Ages	Numbers	
≤45	40	7
>45	66	16
Gender		
Male	63	13
Female	43	11
WHO grade		
I	12	–
II	15	–
III	30	–
IV	49	–
Histopathological classification		
	Astrocytoma	–
IDH status		
	Wildtype	–

### RNA extraction and transcriptome data analysis

Samples (three glioblastoma tissues and three corresponding normal brain tissues) were used to extract total RNA by using TRIzol reagent (Invitrogen) according to the manufacturer’s instructions. The RNA quality was measured with a NanoDrop ND-3300 and verified by gel electrophoresis. Ribo-minus transcriptome libraries were constructed with the TruSeq Ribo Profile Library Prep Kit (Illumina) according to the manufacturer’s protocols. The libraries were then subjected to 151-nt paired-end sequencing, generating a depth of ~ 100 million read pairs, with an Illumina Nextseq 500 system (Novogene). Adapters were first trimmed with cut adapt to obtain clean reads and the remaining reads were aligned to the human genome (hg19) with bowtie2, with one mismatch allowed. Continuous or non-continuous mapped reads were subjected to the following mRNA and lncRNA analyses. Linear expression levels were evaluated with TopHat2 and Cufflinks followed by the annotation references of Refseq. Differentially expressed lncRNAs were identified using strict filtering criteria (| log2(fold change) | ≥ 1.5 and *P* value < 0.01), and (| log2(fold change) | ≥ 1 and P value < 0.01) for mRNAs.

Gene expression profile sets GSE50161 and GSE33331 [43, 44] were downloaded from the Gene Expression Omnibus database (https://www.ncbi.nlm.nih.gov/geo/) [45]. The two datasets were based on GPL570 [HG-U133_Plus_2] Affymetrix Human Genome U133 Plus 2.0 Array. The two datasets were merged to research the expression trend of lncRNAs. GEPIA (Gene Expression Profiling Interactive Analysis) (http://gepia.cancer-pku.cn) [46], a web-based tool that delivers fast and customizable functionalities based on TCGA and GTEx data, was employed to further verify the expression profile of lncRNAs.

### Bioinformation analysis

The Kyoto Encyclopedia of Genes and Genomes (KEGG) database (http://www.genome.jp/kegg/) is widely applicable to systematic analysis of gene functions [47]. Database for annotation, visualization, and integrated discovery (DAVID) is an analytical tool that is used for integrative analysis of large gene lists [48]. In this study, we used DAVID (version 6.8) to perform KEGG pathway enrichment analyses for differentially expressed genes with the following cutoff thresholds: enrichment gene number > 2 and *P* value < 0.05.

### Cell lines and culture conditions

Human glioma cell lines (U251, U87, SHG44, T98G, GOS-3, TJ905, U373) and normal cells (HEB) were obtained from Shanghai Institute of Biochemistry and Cell Biology, Chinese Academy of Sciences (Shanghai, China) and maintained in our lab. All cell lines underwent a mycoplasma contamination test and determined to be mycoplasma-free. All cells were cultivated in high-glucose Dulbecco’s Modified Eagle Medium (DMEM, HyClone) containing 10% FBS (Clark) and stored in an incubator with 5% CO_2_ at a constant temperature of 37 °C.

### RNA extraction and PCR

Total RNA from glioma tissues and cell lines was extracted using TRIzol Reagent (Invitrogen) according to the manufacturer’s protocols, and 1 μg of RNA quantified by a NanoDrop ND-3300 (Thermo Fisher Scientific) was reverse transcribed using GoScript Reverse Transcription System (Promega) with corresponding primers. Real-time PCR analyses were performed with TransStart Top Green qPCR SuperMix (+Dye II) (TransGen) on an ABI Q5 Sequence Detection system (Applied Biosystems); GAPDH was used as an internal control. Bulge-Loop miRNA-specific Primer (RiboBio) was applied to measure miR-7-5p expression according to the manufacturer’s synopsis, and U6 was used as an endogenous control. Relative mRNA and miRNA expression levels were analyzed using the 2-^ΔΔ^Ct method. All primers were synthesized by Sangon Biotech; detailed information is shown in Table S1.

### Nuclear-cytoplasmic fractionation

Nuclear/cytoplasmic fractionation was performed with a Nuclei Isolation Kit (KeyGEN BioTECH) according to the manufacturer’s protocols. Nuclear and cytoplasmic RNA was analyzed by real-time quantitative PCR; U6 was used as the nuclear fraction control, while GAPDH served as the cytoplasmic fraction control.

### Plasmids, siRNAs, and transfection

For LPP-AS2 and EGFR overexpression, full-length LPP-AS2 and EGFR cDNA was amplified and subcloned into pEGFP-C1; the empty vector was used as a negative control. All plasmids were isolated using Endo-free Plasmid DNA Mini Kit I (OMEGA). SiRNAs, miRNA mimics and inhibitors were all obtained from RiboBio. All siRNAs were BLAST searched to ensure that no more than 17-nt matches occurred in the corresponding genomes [49]. SiRNA and plasmid transfection was conducted with Lipofectamine 3000 reagent (Invitrogen) or lipo8000 reagent (Beyotime) in accordance with the manufacturer’s protocol.

### Lentiviral vector construction and stable transfection

Lentiviral constructs of sh-LPP-AS2 was conducted by Hanbio Biotechnology and constructed into SHG44 cell lines. Cells were transfected with lentivirus or negative control virus (NC) in order to select the stably transfected cells. The cells were then treated with puromycin (2 μg/mL) (Solarbio) for 2 weeks. GFP-positive cells were selected as sh-LPP-AS2 and sh-NC stably transfected cells and validated by real-time quantitative PCR.

### Tumor xenograft model

Female BALB/c nude mice (aged 4–5 weeks, 18–20 g) were purchased from Vital River Laboratory Technology, and reared in laminar airflow cabinets under specific pathogen-free conditions. Subsequently, 1 × 10^7^ cells stably transfected with sh-LPP-AS2 or sh-control were suspended in 0.1 mL PBS and 0.1 mL Matrigel substrate and injected subcutaneously into the armpit regions of the mice. Tumor volumes were measured every 3 days and calculated using the following formula: volume (cm^3^) = (length × width^2^)/ 2. Bioluminescent imaging was performed using IVIS Lumina LT Series III Imaging System (IVIS Lumina) with administration of D-luciferin (150 mg/kg i.v.). The mice were sacrificed after 18 days post-injection, and the tumors were gathered for subsequent analysis. The animal studies were approved by the Institutional Animal Care and Use Committee of the First Affiliated Hospital of USTC.

### RNA pull-down with biotinylated antisense oligonucleotides

RNA pull-down with 5′-biotinylated AS oligos was used a previously described method [50]. Cells were cross-linked in a UV cross-linker (UVP) at a strengthen of 200-mJ. The cells were granulated and resuspended in RIPA buffer (50 mM Tris-Cl, pH 8.0, 150 mM NaCl, 5 mM EDTA, 1% NP-40, 0.1% SDS, 1 mM DTT, 1× protease-inhibitor cocktail (Roche), and 0.1 U/μL RNase inhibitor) for 10 min on ice and then harvested and sonicated for 10 min. Cell debris in the lysate was removed by centrifugation at 13,000 g for 20 min. Subsequently, biotinylated AS oligonucleotides (100 pmol) or Scramble oligos (as control) were added to the supernatant at 4 °C for 2 h. M-280 Streptavidin Dynabeads (Thermo) were washed three times in RIPA buffer and then blocked with 500 ng/μL yeast total RNA and 1 mg/mL BSA for 1.5 h at room temperature. Subsequently, the beads were washed three times again in RIPA buffer and half of washed/blocked beads was added per 100 pmol of biotin-DNA oligonucleotides. Then, the mixture was rotated for 4 h at 4 °C. Beads were captured using magnets (Life Technologies) and washed three times with RIPA buffer supplemented with 500 mM NaCl. RNAs and proteins were extracted from the beads and used for further analysis.

### RNA immunoprecipitation assay (RIP)

Briefly, the 10^7^ cells were washed with cold 1× PBS three times and irradiated in a UV cross-linker (400 mJ/cm^2^, 2 min). The whole cells were harvested in ice-cold lysis buffer (10 mM HEPES, pH 7.4, 200 mM NaCl, 30 mM EDTA, 0.5% Triton-X 100, 100 units/mL RNasin Plus RNase Inhibitor (Promega), 1.5 mM DTT, 1× protease-inhibitor cocktail (Roche)) and sonicated for 5 min with an ultrasonic disruptor (SONICS). The cell suspension was centrifuged at 13,000 g for 15 min at 4 °C and the supernatant was collected. A 200-μL sample of the supernatant was saved as input. Subsequently, anti-AGO2 (Proteintech) or IgG (Sangon Biotech) antibody was added into the cell suspension and incubated for 2 h at 4 °C. Protein G Dynabeads (Life Technology) suspension was washed three times with RIPA buffer and then blocked with 500 ng/μL yeast total RNA and 1 mg/mL BSA for 1.5 h at room temperature. The beads were washed three times again in RIP buffer and then added into cell suspension for binding for at least 4 h at 4 °C. The antibody–protein G bead complexes were washed five times with lysis buffer and digested with 30 μg of proteinase K at 65 °C for 1 h. Finally, the immunoprecipitated RNA was purified and detected by quantitative real-time PCR. Antibody validation is provided on the manufacturers’ websites.

### Chromatin immunoprecipitation assay (ChIP)

ChIP was carried out as previously described, with modifications [51]. Cells were cross-linked in a UV cross-linker (UVP) at 200-mJ strength. Cell granules were lysed in 1 mL of SDS lysis buffer (1% (w/v) SDS, 10 mM EDTA, and 50 mM Tris-HCl, pH 8.1, Complete protease-inhibitor cocktail (Roche)) after being washed with cold-PBS three times and then were incubated for 20 min on ice. The cell mixture was sonicated for 5 min with an ultrasonic disruptor (SONICS) to obtain up to 500-bp DNA fragments. A 100-μL sample of the supernatant was saved as input. The chromatin solution was immunoprecipitated with an antibody to c-MYC (Proteintech, validation provided on the manufacturer’s website) or IgG, and the mixture was rotated for 2 h at room temperature. Then, Protein G Dynabeads (Life Technology) suspension was washed with lysis buffer three times and blocked with 500 ng/μL yeast total RNA and 1 mg/mL BSA for 1.5 h at room temperature. The beads were washed three times again in lysis buffer and then added into cell suspension for binding for at least 4 h at room temperature. The beads were collected and digested with proteinase K for 1 h at 45 °C, and the DNA was extracted by an Endo-free Plasmid DNA Mini Kit (OMEGA). The eluted DNA was subjected to quantitative real-time PCR using the corresponding PCR primers to detect the enriched genomic DNA region.

### Dual luciferase reporter assay

Approximately 1 × 10^4^ human U251 cells were co-transfected with 50 nM empty pmirGLO-NC, pmirGLO-LPP-AS2-wt (or pmirGLO-EGFR-wt) or pmirGLO-LPP-AS2-mut (or pmirGLO-EGFR- mut) (RiboBio) and 50 nM miR-7-5p mimics or miR-NC using Lipofectamine 3000 (Invitrogen) according to the manufacturer’s protocols. The firefly luciferase gene in the vector pmirGLO-control (Promega) was used as the endogenous control to detect transfection efficiency. Firefly and Renilla luciferase activities were measured with a Dual-Luciferase Reporter Assay System (Promega) after 48 h transfection. Firefly luciferase activity was normalized to the corresponding Renilla luciferase activity. Experiments were performed in triplicate, and the data are represented as the mean ± SD.

### Cell proliferation assay

Proliferation of U251 and SHG44 glioma cells was measured by using the Cell Counting Kit-8 (Biosharp) assay. A total of 4 × 10^3^ U251 or SHG44 glioma cells/well was seeded in 96-well plates and incubated in a 5% CO_2_ atmosphere at 37 °C. 10 μL of CCK-8 solution was added to each well at 0, 24, 48 and 72 h after cell transfection. After incubation for 3 h, the absorbance value per well was determined with an ultraviolet spectrophotometer at 490 nm.

### Apoptosis detection by flow cytometry and TUNEL assay

U251 and SHG44 cells (2 × 10^5^ cell/well) were seeded into 6-well plates and harvested at 48 h post-transfection. The cells were washed with PBS, centrifuged twice and resuspended in Annexin-V binding buffer. Annexin V-FITC/PI staining was performed according to the manufacturer’s protocols and the apoptosis rate was analyzed by Gallios flow cytometry (BECKMAN COULTER). In addition, cell apoptosis was measured by One-Step TUNEL Apoptosis Kit (RiboBio) according to the manufacturer’s introductions.

### Cell migration and invasion assay

Cell migration and invasion abilities were detected by Transwell assays. For cell migration detection, transfected U251 or SHG44 cells (1 × 10^4^ cells) were harvested after 24 h transfection and resuspended in 100 μL serum-free medium. Then, the cells were seeded into the upper chamber of a Transwell assay insert (Millipore), and 700 μL 10% FBS medium was added to the lower chamber. After incubation at 37 °C for 48 h, the cells on the lower side were washed three times with PBS, fixed in 4% paraformaldehyde for 20 min, and stained with crystal violet solution for 15 min. Five random fields were chosen to count stained cells for statistics under an inverted microscope (Olympus) and photographs were taken. For the invasion assay, Transwell chambers were coated with Matrigel for 1 h at 37 °C. The transfected cells (1 × 10^4^ cells) were resuspended in 100 μL serum-free medium and seeded into the upper chamber. Then 700 μL 10% FBS medium was added to the lower chamber. After a 48 h incubation period, the invasive ability was evaluated as mentioned previously for the cell migration assay.

### Wound-healing assay

Transfected U251 and SHG44 cells (5 × 10^4^ cells) were seeded into each side of a Culture-Insert 2 Well (Ibidi), and the μ-Dish was filled with 2 mL 2% FBS medium. Images of the different stages of wound healing were photographed via microscopy at 0, 12 and 24 h. Relative wound-healing rates were calculated by using CellSense Standard software (Olympus) and each experiment was performed in triplicate.

### Colony formation assay

For colony formation assays, transfected U251 or SHG44 cells were harvested after 24 h transfection and 300 cells were inoculated into 6-well plates. After incubation at 37 °C for 14 days, the colonies were fixed with 4% paraformaldehyde and stained with crystal violet solution.

### Immunohistochemistry (IHC)

Immunohistochemistry used a previously described method [52]. Dissected tumors from the mouse model were fixed overnight in formalin solution, dehydrated in ethanol, embedded in paraffin, and cut into 5 μm sections. Then, the specimens were treated with xylene and ethanol to remove paraffin. The slides were blocked with 5% normal goat serum and incubated with anti-Ki67, anti-MMP-9 or anti-EGFR antibodies overnight at 4 °C and washed three times with PBS. After incubation with an HRP-conjugated secondary antibody, the sections were counterstained with hematoxylin. The average integral optical density of each positively stained slide was measured using ImageJ software. Three fields were chosen randomly from each section for measurement.

### Western blotting

For western blotting, samples were separated by 10% SDS-PAGE and electrophoretically transferred to PVDF membranes (Millipore). The membranes were processed according to the ECL western blotting protocol (GE Healthcare) and scanned with Amersham Imager 680 (GE Healthcare). The following primary antibodies were used: anti-EGFR (BBI, D260292); anti-PI3K (BBI, D155308); anti-AKT (Proteintech, 10,176–2-AP); anti-p-PI3K Tyr458 (Cell Signaling Technology, 4228S); anti-p-AKT Ser473 (Proteintech, 66,444–1-Ig); anti-c-MYC (Proteintech, 10,828–1-AP) and anti-β-actin (BBI, D110001). The anti-β-actin antibody was used as an endogenous control for normalization. Antibody validation is provided on the manufacturers’ websites.

### Data deposition

RNA-sequencing data have been deposited in the Gene Expression Omnibus (GEO) database under accession number GSE153692.

### Statistical analysis

All experiments were performed in triplicate; data were analyzed using SPSS (version 23.0) or GraphPad Prism software (version 7.0), and the results are presented as the mean ± SD. Student’s t tests were used to calculate *P* values, as indicated in the figure legends, while one way analysis of variance (ANOVA) was used for multiple data groups. Correlations between lncRNA LPP-AS2 and miR-7-5p as well as EGFR in human specimens were analyzed by Spearman’s rank test. Survival curves were generated using the Kaplan-Meier method and log-rank tests. P value < 0.05 was considered statistically significant.

## Results

### LncRNA LPP-AS2 is significantly upregulated in glioma tissues and transcriptionally regulated by c-MYC

**Fig. 1:**
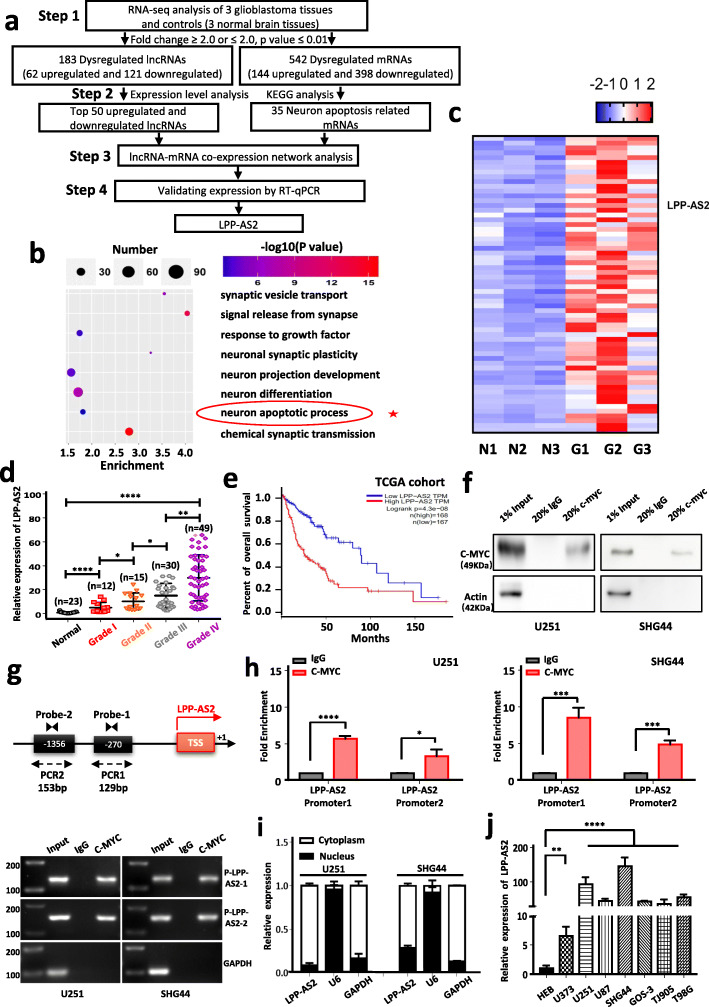
RNA-seq analysis reveals that LPP-AS2 is significantly upregulated in glioblastoma tissues and transcriptionally regulated by c-MYC. **a** Flow diagram describes the steps for identifying and validating lncRNAs in glioblastoma. **b** The KEGG pathway analysis of differentially expressed lncRNAs. **c** Heatmaps of top 50 lncRNAs that were differentially expressed between glioblastoma and normal brain tissues. Upregulated lncRNAs are shown in red and downregulated lncRNAs are shown in blue. **d** Relative expression of LPP-AS2 was significantly elevated in (12 Grade I, 15 Grade II, 30 Grade III and 49 Grade IV) glioma tissues compared with 23 normal brain tissues. **e** Kaplan-Meier analysis of overall survival in glioma patients with low (*n* = 167) and high (*n* = 168) level of LPP-AS2. *P* value was calculated by Mantel-Cox log rank test. **f** Pulldown of LPP-AS2 in Chromatin immunoprecipitation assay with c-MYC. Results of western blots suggesting c-MYC could effective pulldown of LPP-AS2, and β-actin as the negative control. **g** Illustration of c-MYC bound to the promoter regions of LPP-AS2. Transcription start site (TSS) was designated as + 1. The putative binding sites were listed. **h** C-MYC significantly enhanced the fold enrichment of LPP-AS2 probe compared with IgG in U251 and SHG44 cells. **i** Relative level of LPP-AS2 in the nuclear and cytoplasmic fractions of U251 and SHG44 cells. **j** Relative expression of LPP-AS2 in seven glioma cell lines and HEB by RT-qPCR. Error bars, s.e.m. from three independent experiments. *P < 0.05; **P < 0.01; ***P < 0.001; ****P < 0.0001 (Student’s t test)

To investigate expression profiles of lncRNAs and mRNAs in glioma, high-throughput RNA sequencing was performed in 3 tissues from glioblastoma patients (diagnosed by pathological biopsy) and 3 normal brain tissues from craniocerebral trauma patients. To ensure the authenticity and validity of the RNA-seq data, we examined the expression levels of some previously reported mRNAs such as TGFB2, HOXB3, FOXM1 and CD44, and lncRNAs such as H19, HCP5, PART-1 and MEG3 in our data (Figure S1a) [53–60]. The same pattern of these positive controls in glioma made our RNA-seq results more convincing. Hierarchical cluster analysis was applied to reveal differential expression of lncRNAs and mRNAs in glioblastoma tissues and controls (Figure S1b). Volcano plots were also performed for all expressed mRNAs in glioblastoma tissues and controls (Figure S1c). A total of 183 lncRNAs were identified as significantly dysregulated in glioblastoma, of which 62 were upregulated and 121 downregulated (step 1 in Fig. 1a). Further validation by RT-qPCR revealed SMIM30, SNORA53 and LINC01354 were significantly upregulated and EFEMP2 markedly downregulated in 15 glioblastoma samples compared to 6 controls samples (Figure S2a). In addition, 542 differentially expressed mRNAs were also identified, with 144 upregulated and 398 downregulated (step 1 in Fig. 1a). To validate these results, 6 mRNAs were randomly selected for RT-qPCR analysis using 15 glioblastoma samples and 6 normal tissues. TOP2A, COL4A1 and PXDN were significantly upregulated and ANK3, ARRB1 and ANO4 markedly downregulated in glioblastoma compared to controls (Figure S2b). To identify pivotal lncRNAs involved in glioblastoma, a co-expression network analysis was carried out between 35 mRNAs in the neuron apoptotic process (Fig. 1b) as predicted by KEGG pathway analysis and the top 50 lncRNAs (Fig. 1c). The network revealed that LPP-AS2 might play crucial roles in tumor apoptosis (Figure S2c). Thus LPP-AS2 was selected for further investigation.

Notably, we found the expression of LPP-AS2 was markedly increased in 106 glioma tissues (12 Grade I, 15 Grade II, 30 Grade III and 49 Grade IV) compared with 23 normal brain tissues by RT-qPCR (Fig. 1d). To reduce false positives, GSE50161 and GSE33331, microarray profiles based on the GPL570 platform, were downloaded from the GEO database, together with the GEPIA (Gene Expression Profiling Interactive Analysis) tool to further validate the observed upregulated expression of LPP-AS2 (Figure S2d, e). Especially, increased levels of LPP-AS2 was significantly correlated with advanced grade in glioma patients (Fig. 1d). In addition, Kaplan-Meier analysis of GEPIA demonstrated that patients with higher LPP-AS2 expression were more likely to have poor overall survival (Fig. 1e).

It has been reported that c-MYC inhibited the expression of LPP-AS2 in colorectal cancer [27]. To explore whether c-MYC could affect LPP-AS2 expression in glioma, the promoter sequence analysis tool (USCS) was performed to search the 2000 bp upstream of LPP-AS2; based on the results, the LPP-AS2 promoter region is predicted to harbor two c-MYC binding sites. Chromatin immunoprecipitation (ChIP) followed by RT-PCR and RT-qPCR assays were employed to verify the putative correlation between the LPP-AS2 promoter region and c-MYC (Fig. 1f-h). Meanwhile, knockdown of c-MYC significantly decreased the expression level of LPP-AS2 (Figure S2f). These results supported the claim that c-MYC directly binds to the two putative chromatin fragment of promoter regions of LPP-AS2 and then regulate the transcription of LPP-AS2 (Fig. 1f-h). Collectively, these data suggested that the c-MYC transcriptionally regulated lncRNA LPP-AS2 was highly expressed in glioma tissues, and might be a promising indicator of glioma prognosis.

### LPP-AS2 promotes glioma progression in vitro and in vivo

RT-qPCR analysis of nuclear and cytoplasmic RNAs was carried out to show that LPP-AS2 was preferentially localized in the cytoplasm (Fig. 1i). Besides, expression of LPP-AS2 was significantly elevated in seven glioma cell lines (U251, SHG44, T98G, U373, U87, GOS-3, and TJ905) compared to the normal cell line HEB and was higher in the U251 and SHG44 cell lines (Fig. 1j). Thus, we selected the U251 and SHG44 cell lines for subsequent functional analyses.

**Fig. 2:**
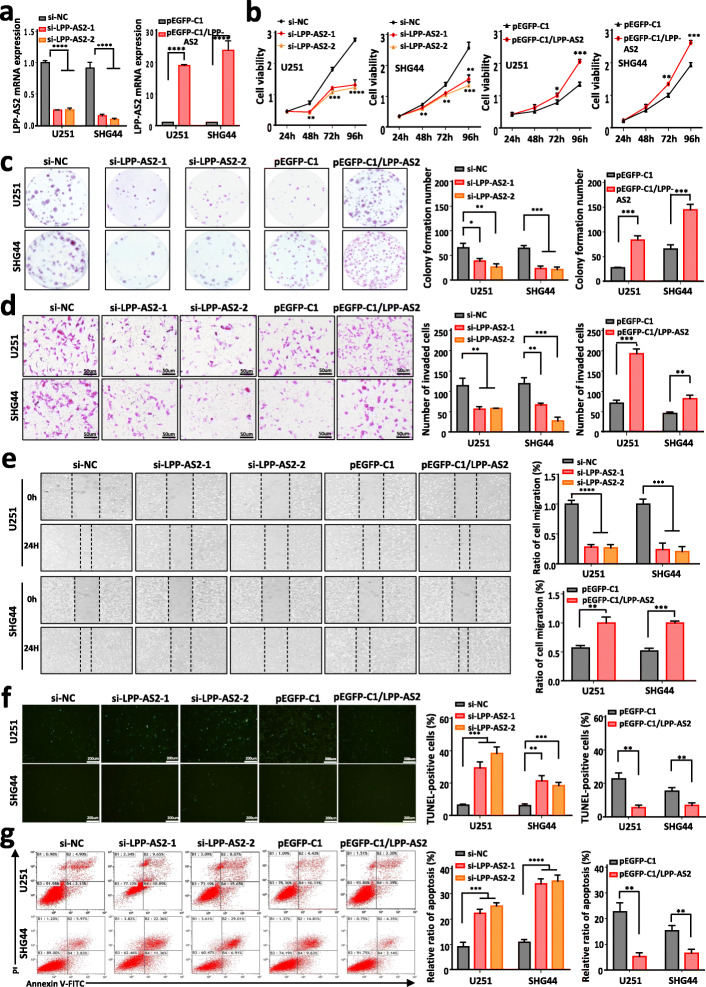
LPP-AS2 promotes glioblastoma progression in vitro. **a** Relative expression of LPP-AS2 in U251 and SHG44 cells transfected with siRNAs/overexpression plasmids. **b** CCK-8 assays in U251 and SHG44 cells with silencing/overexpression of LPP-AS2. **c** Colony formation assays in U251 and SHG44 with silencing/overexpression of LPP-AS2. **d** Transwell assays in U251 and SHG44 cells with silencing/overexpression of LPP-AS2. Representative staining images are presented (bar = 50 μm). **e** Wound healing assays of U251 and SHG44 cells with silencing/overexpression of LPP-AS2. **f** TUNEL assays in U251 and SHG44 cells with silencing/overexpression of LPP-AS2 (bar = 200 μm). **g** Flow cytometry analysis in U251 and SHG44 cells with silencing/overexpression of LPP-AS2. Data represent the mean ± s.e.m. of three independent experiments. *P < 0.05; **P < 0.01; ***P < 0.001; ****P < 0.0001 (Student’s t test)

To explore the biological functions of LPP-AS2, two independent small interfering RNAs (siRNAs) against LPP-AS2 were designed, which produced effective knockdown of the expression of LPP-AS2 in U251 and SHG44 cells (Fig. 2a). We also constructed an overexpression plasmid and successfully overexpressed LPP-AS2 in U251 and SHG44 cells (Fig. 2a). Next, CCK8 and colony formation assays demonstrated that depletion of LPP-AS2 markedly inhibited cell proliferation, whereas overexpression of LPP-AS2 expression facilitated cell viability (Fig. 2b and c). According to Transwell and wound-healing assays, knockdown of LPP-AS2 significantly suppressed cell invasion and migration capacities, whereas upregulation of LPP-AS2 exhibited the opposite effects (Fig. 2d and e). We observed same effects in the results of Transwell and wound-healing assays even though treated with Ara-C, which was usually used to inhibit cell proliferation (Figure S3a-c). In parallel, apoptosis was analyzed following LPP-AS2 silencing or overexpression by TUNEL assays and flow cytometry. As expected, knockdown of LPP-AS2 induced cell apoptosis, but upregulation of LPP-AS2 inhibited cell apoptosis (Fig. 2f and g).

**Fig. 3:**
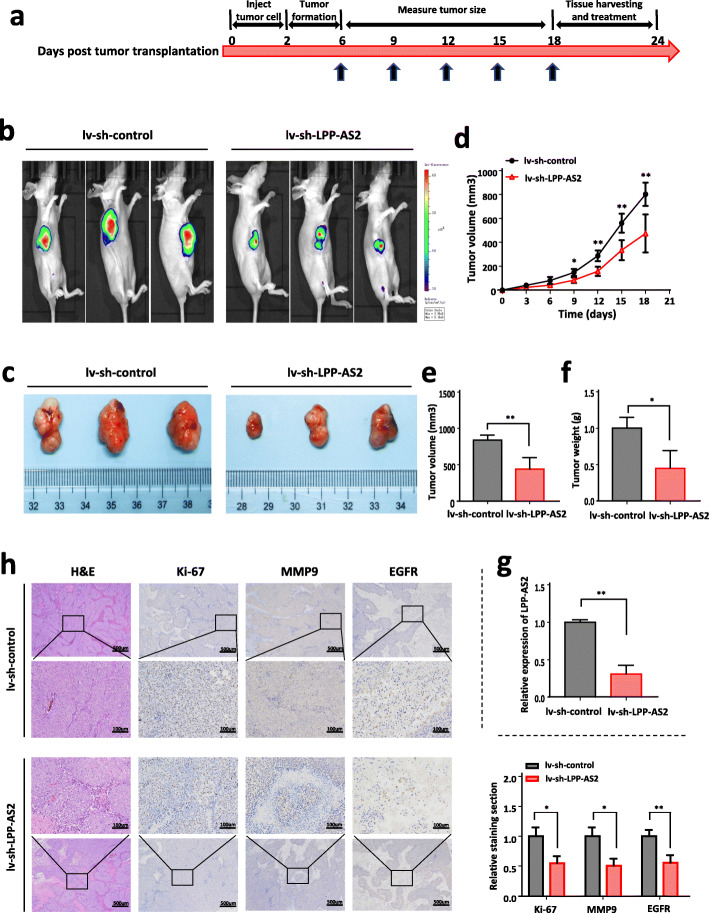
Downregulation of LPP-AS2 suppresses glioblastoma progression in vivo. **a** Schematic diagram of the entire experimental process. The divergent arrows suggest the different stages (first, inject tumor cells; second, tumor formation; third, measure tumor size; fourth, tissue harvesting and treatment). **b**, **c** Representative images of tumor formation of the lv-sh-control group and lv-sh-LPP-AS2 group. The dissected tumors from two groups were photographed. **d** Tumor volume was measured every 3 days. **e** Volumes of xenograft tumors in lv-sh-LPP-AS2 group and lv-sh-control group. **f** Weights of xenograft tumors in lv-sh-LPP-AS2 group and lv-sh-control group. **g** Relative expression of LPP-AS2 in Xenograft tissues were measured by RT-qPCR. **h** IHC staining revealed that transfection of lv-sh-LPP-AS2 contributed to decreased Ki-67, MMP9 and EGFR expression in the subcutaneous tumors. Data represent the mean ± s.e.m. of three independent experiments. *P < 0.05; **P < 0.01; (Student’s t test)

To evaluate the regulatory roles of LPP-AS2 in tumor formation in vivo, a nude mice xenograft tumor model was constructed. SHG44 stable cells were established with lentivirus to downregulate LPP-AS2, and 1 × 10^7^ SHG44 cells with stable expression were subcutaneously injected into BALB/c nude mice (two groups, *n* = 3 each group). The procedure of the animal study is as follows (Fig. 3a). After injection, smaller tumor sizes and weights were observed in the LV-sh-LPP-AS2 group than in the LV-sh-control group (Fig. 3b-f). Expression of LPP-AS2 was effectively downregulated in the LV-sh-LPP-AS2 group (Fig. 3g). Furthermore, H&E and immunohistochemistry staining demonstrated that the expression of Ki-67, matrix metalloprotease (MMP-9) and epidermal growth factor receptor (EGFR) was reduced upon LPP-AS2 downregulation in dissected tumors (Fig. 3h). These findings suggested that LPP-AS2 promoted cell proliferation, migration, invasion and inhibited apoptosis of glioma cells in vitro and in vivo.

### LPP-AS2 facilitates gliomas progression by enhancing EGFR expression

**Fig. 4:**
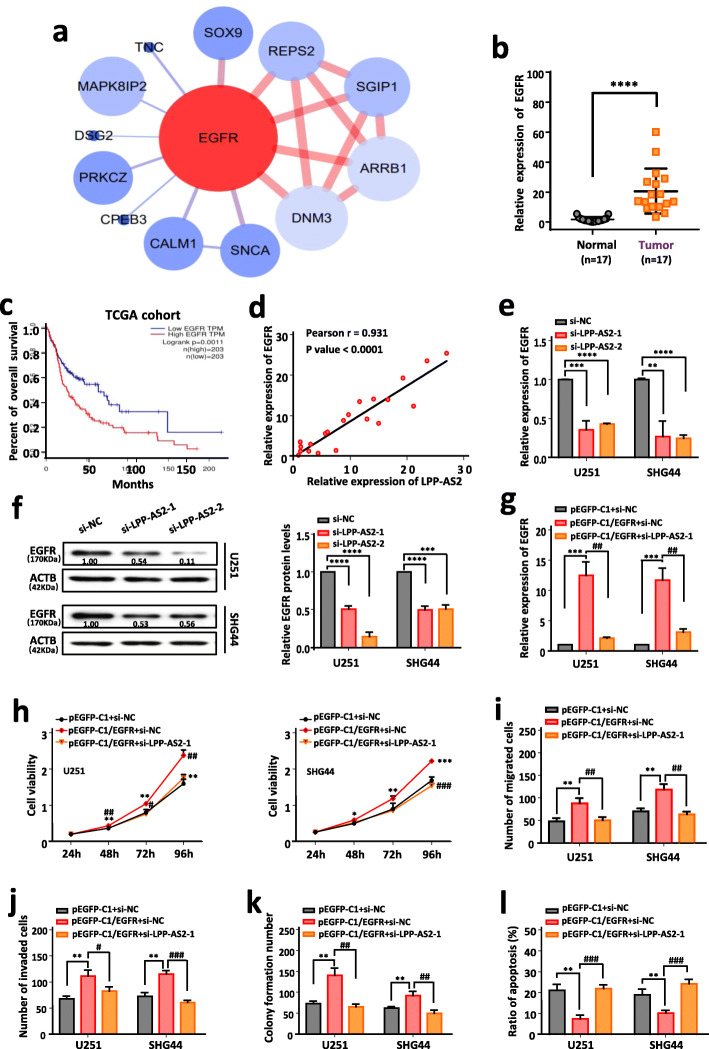
LPP-AS2 facilitates tumor progression by regulating expression of EGFR. **a** Corresponding mRNAs in co-expression analysis network of LPP-AS2. Upregulated mRNAs are shown in red and downregulated mRNAs are shown in blue. **b** Relative expression of EGFR in 17 glioblastoma tissues and 17 normal brain tissues by RT-qPCR. **c** Kaplan-Meier analysis of overall survival in glioma patients with low (*n* = 203) and high (*n* = 203) level of EGFR. *P* value was calculated by Mantel-Cox log rank test. **d** The Pearson correlation between LPP-AS2 level and EGFR level was measured in the same set of glioblastoma tissues. The ΔCt values (normalized to GAPDH) were subjected to Pearson correlation analysis (*R* = 0.93, *P* < 0.0001). **e** Knockdown of LPP-AS2 significantly inhibited EGFR expression in U251 and SHG44 cells. **f** Protein level of EGFR in U251 and SHG44 cells after treated with siRNAs of LPP-AS2. **g** Relative expression of EGFR in U251 and SHG44 cells transfected with pEGFP-C1/EGFR alone or co-transfection with siRNAs of LPP-AS2. **h** CCK8 assays in U251 and SHG44 cell treated with pEGFP-C1/EGFR alone or co-transfection of si-LPP-AS2–1. **i**, **j** Histogram of migration and invasion assays in U251 and SHG44 cells treated with pEGFP-C1/EGFR alone or co-transfection of si-LPP-AS2–1. **k** Colony formation assay in U251 and SHG44 cells treated with pEGFP-C1/EGFR alone or co-transfection of si-LPP-AS2–1. **l** Histogram of cell apoptosis rate in U251 and SHG44 cells treated with pEGFP-C1/EGFR alone or co-transfection of si-LPP-AS2–1. Error bars, s.e.m. from three independent experiments. *P < 0.05; **P < 0.01; ***P < 0.001; ****P < 0.0001; ^#^P < 0.05; ^##^P < 0.01; ^###^*P* < 0.001; ^####^*P* < 0.0001 (Student’s t test)

To elucidate the potential molecular mechanisms of LPP-AS2 in glioma initiation and progression, we first detected the expression level of the parent gene LPP. It turned out that overexpression and knockdown of LPP-AS2 had no effect on LPP expression (Figure S4a, b). Therefore, we constructed a co-expression analysis network of LPP-AS2 and corresponding mRNAs according to the data of high-throughput RNA sequencing. EGFR was the top predicted co-expressed mRNA and was markedly upregulated in glioma tissues (Fig. 4a). We further confirmed upregulation of EGFR in 17 glioblastoma tissues compared to normal controls (Fig. 4b). Survival analysis of TCGA database revealed that patients with high expression of EGFR were significantly associated with poor prognosis (Fig. 4c). Moreover, correlation analysis based on RT-qPCR results showed a positive correlation between the expression level of EGFR and LPP-AS2 (Fig. 4d). Additionally, expression of EGFR was significantly increased in seven glioma cell lines (U251, SHG44, T98G, U373, U87, GOS-3, and TJ905) compared to the normal cell line HEB (Figure S4c). The results of RT-qPCR and western blot suggested that LPP-AS2 knockdown significantly reduced the mRNA and protein levels of EGFR, whereas EGFR mRNA and protein levels were upregulated by overexpressed LPP-AS2 (Fig. 4e, f and Figure S4d, e). Taken together, EGFR was considered a major candidate target of LPP-AS2.

Furthermore, the findings of rescue functional experiments revealed that cell proliferation, migration, invasion, and colony formation abilities were promoted/inhibited by overexpression/knockdown of EGFR and were reversed by silencing/increasing LPP-AS2 expression (Fig. 4g-k and Figure S5a-e). Restoration/downregulation of LPP-AS2 expression partially rescued the suppressive/accelerated effects of EGFR knockdown/overexpression on glioma cell apoptosis (Fig. 4l and Figure S5f). All these findings illustrated that LPP-AS2 plays an oncogenic role in glioma progression through EGFR.

### LPP-AS2 functions as a ceRNA and competitively absorbs miR-7-5p in glioma cells

**Fig. 5:**
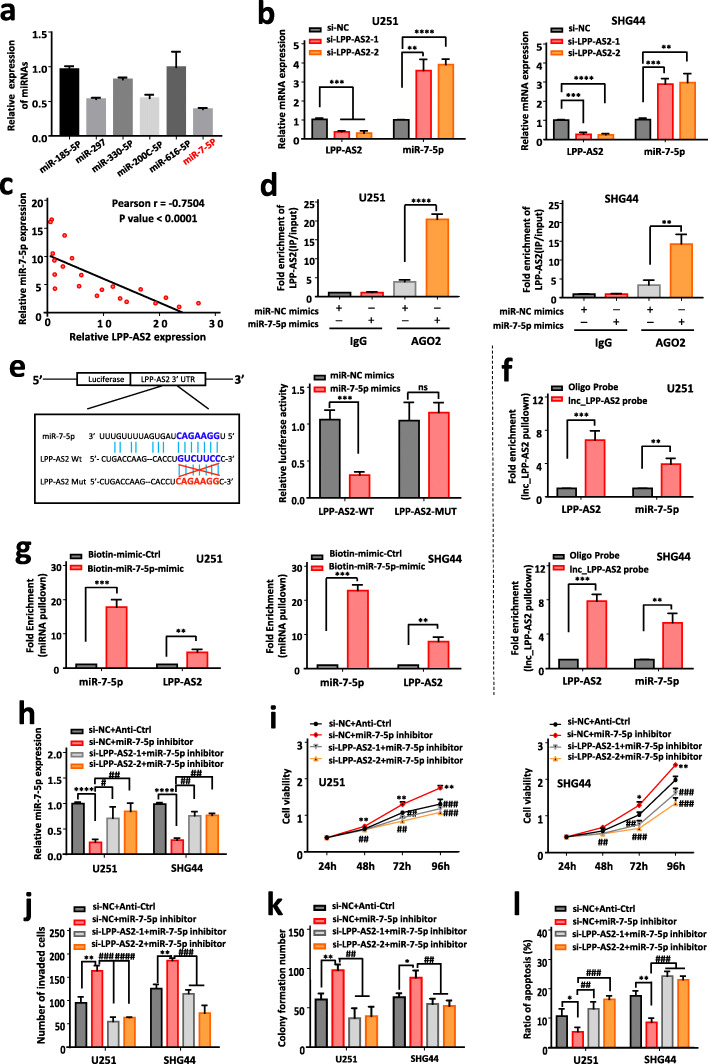
LPP-AS2 regulates glioblastoma progression by directly bind with miR-7-5p. **a** Relative expression of miRNAs align to LPP-AS2. **b** Knockdown of LPP-AS2 significantly upregulated the expression of miR-7-5p in U251 and SHG44 cells. **c** The Pearson correlation between LPP-AS2 level and miR-7-5p level was measured in the same set of glioblastoma tissues. The ΔCt values (normalized to GAPDH) were subjected to Pearson correlation analysis (*R* = -0.7504, *P* < 0.0001). **d** Ago2 protein immunoprecipitation was used to detect the fold enrichment of LPP-AS2 in U251 and SHG44 cells. **e** Schematic representation of the potential binding sites between LPP-AS2 and miR-7-5p. Luciferase report activity of constructed plasmids (LPP-AS2-WT or LPP-AS2-MUT) in 293 T cells co-transfected with miR-7-5p mimics or miR-NC mimics. **f** Lnc_LPP-AS2 pulldown was used to measure the fold enrichment of LPP-AS2 and miR-7-5p in U251 and SHG44 cells. **g** Enrichment of LPP-AS2 in U251 and SHG44 cells after treated with biotinylated miR-7-5p. **h** RT-qPCR analysis of miR-7-5p expression in U251 and SHG44 cells transfected with miR-7-5p inhibitor alone or co-transfection with siRNAs of LPP-AS2. **i** CCK8 assays in U251 and SHG44 cell treated with miR-7-5p inhibitor alone or co-transfection with siRNAs of LPP-AS2. **j** Histogram of invasion assays in U251 and SHG44 cells treated with miR-7-5p inhibitor alone or co-transfection with siRNAs of LPP-AS2. **k** Histogram of colony formation assays in glioma cells treated with miR-7-5p inhibitor alone or co-transfection with siRNAs of LPP-AS2. **l** Histogram of flow cytometry assays in U251 and SHG44 cells treated with miR-7-5p inhibitor alone or co-transfection with siRNAs of LPP-AS2. Data represent the mean ± s.e.m. of three independent experiments. ns (no significance); *P < 0.05; **P < 0.01; ***P < 0.001; ****P < 0.0001; ^#^*P* < 0.05; ^##^*P* < 0.01; ^###^*P* < 0.001; ^####^*P* < 0.0001 (Student’s t test)

Recently, a number of cytoplasmic lncRNAs have been shown to function as sponges for miRNAs in order to regulate downstream targets [61–64]. As shown in Fig. 1i, LPP-AS2 predominantly localized in the cytoplasm, and we thus hypothesized that LPP-AS2 acts as a competing endogenous (ce) RNA based on these findings. To validate this hypothesis, we used an online bioinformatic tool (lncRNASNP2.0) to predict miRNAs able to bind with LPP-AS2 and found that LPP-AS2 contains potential complementary binding sequences for miR-7-5p, miR-200c-5p, miR-297, miR-185-5p, miR-616-5p and miR-330-5p seed regions. The results of RT-qPCR suggested miR-7-5p to be the most downregulated miRNA (Fig. 5a). Indeed, miR-7-5p showed obvious downregulation in 17 glioblastoma tissues compared to normal control samples (Figure S6a), and miR-7-5p expression was significantly downregulated in multiple glioma cell lines including U251 and SHG44 cells (Figure S6b). Additionally, knockdown of LPP-AS2 significantly increased the expression level of miR-7-5p (Fig. 5b). Overexpressed LPP-AS2 reduced the expression level of miR-7-5p (Figure S6c), but had no effect on LPP-AS2 expression when the level of miR-7-5p was altered by miR-7-5p mimics or inhibitor (Figure S6d). Correlation analysis indicated a negative correlation between expression of LPP-AS2 and miR-7-5p (Fig. 5c). As Ago2 is a crucial component of the RNA-induced silencing complex (RISC) [58], we performed an Ago2 immunoprecipitation assay in U251 and SHG44 cells, and determined that LPP-AS2 can serve as a platform for Ago2 and miR-7-5p (Fig. 5d). We then employed dual luciferase reporter assays to validate direct binding of miR-7-5p with LPP-AS2. The results demonstrated that miR-7-5p overexpression distinctly suppressed the luciferase activity of the LPP-AS2-WT group, but without statistical changes in the LPP-AS2-MUT group (Fig. 5e). Besides, in an RNA pull-down assay, biotin-coupled LPP-AS2 successfully pulled down miR-7-5p, and biotin-labelled miR-7-5p also pulled down LPP-AS2 (Fig. 5f and g). Taken together, all these findings indicated that LPP-AS2 acted as a molecular sponge for miR-7-5p in glioma cells.

It is widely reported that miR-7-5p exerts important regulatory roles in a variety of cancers [65, 66]. As shown in Figure S7, miR-7-5p markedly inhibited proliferation and invasion as well as the migration and colony formation abilities of glioma cells. These results suggested that miR-7-5p functions as a suppressor in glioma tumorigenesis. To further verify the cellular phenotype caused by binding of LPP-AS2 with miR-7-5p, we performed rescue functional studies and observed that miR-7-5p mimics suppressed the viability, invasion, migration and colony formation abilities of glioma cells stimulated by overexpression of LPP-AS2. In contrast, an miR-7-5p inhibitor rescued the proliferation, invasion, migration and colony formation abilities of glioma cells with downregulated LPP-AS2 expression (Fig. 5h-k and Figure S8a-d). Moreover, the miR-7-5p inhibitor reversed the cell apoptosis induced by LPP-AS2 knockdown, and transfection of miR-7-5p mimics distinctly reversed the apoptosis suppression occurring with LPP-AS2 overexpression (Fig. 5l and Figure S8e). All of these results indicated that LPP-AS2 functioned as an oncogene via miR-7-5p.

### LPP-AS2 decoys miR-7-5p to upregulate the expression of EGFR

**Fig. 6:**
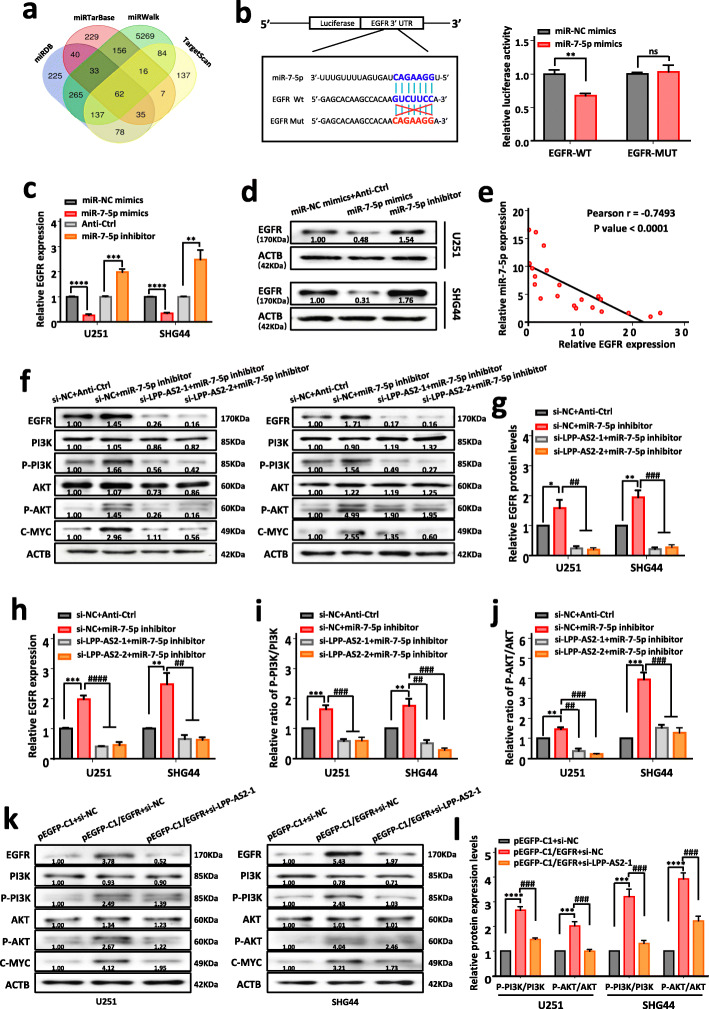
LPP-AS2 decoys miR-7-5p to regulate the expression of EGFR and its downstream PI3K/AKT/c-MYC signaling pathway. **a** A total of 62 targeted mRNAs of miR-7-5p from four online databases (miRDB, miRTarBase, miRWalk, TargetScan). **b** Schematic representation of the potential binding sites between miR-7-5p and EGFR. Luciferase reporter activity of constructed plasmids (EGFR-WT or EGFR-MUT) in 293 T cells co-transfected with miR-7-5p mimics or miR-NC mimics. **c** Relative expression of EGFR in U251 and SHG44 cells transfected with miR-7-5p mimics or miR-7-5p inhibitor. **d** Protein level of EGFR in U251 and SHG44 cells transfected with miR-7-5p mimics or miR-7-5p inhibitor. **e** The Pearson correlation between EGFR level and miR-7-5p level was measured in the same set of glioblastoma tissues. The ΔCt values (normalized to GAPDH) were subjected to Pearson correlation analysis (*R* = -0.7493, *P* < 0.0001). **f** Western blotting analysis of EGFR, PI3K, AKT, p-PI3K, p-AKT, c-MYC in U251 and SHG44 cells treated with miR-7-5p inhibitor alone or co-transfection with siRNAs of LPP-AS2. **g** Histogram of EGFR protein levels in U251 and SHG44 cells treated with miR-7-5p inhibitor alone or co-transfection with siRNAs of LPP-AS2. **h** EGFR mRNA expression in U251 and SHG44 cells treated with miR-7-5p inhibitor alone or co-transfection with siRNAs of LPP-AS2. **i**, **j** Ratio of p-PI3K/PI3K and p-AKT/AKT in U251 and SHG44 cells. **k** Western blotting analysis of EGFR, PI3K, p-PI3K, AKT, p-AKT and c-MYC in U251 and SHG44 cells treated with pEGFP-C1/EGFR plasmids alone or co-transfection with si-LPP-AS2–1. **l** Changes of p-PI3K/PI3K and p-AKT/AKT in U251 and SHG44 cells treated with pEGFP-C1/EGFR plasmids alone or co-transfection of si-LPP-AS2–1. Data represent the mean ± s.e.m. of three independent experiments. ns (no significance); *P < 0.05; **P < 0.01; ***P < 0.001; ****P < 0.0001; ^##^*P* < 0.01; ^###^*P* < 0.001; ^####^*P* < 0.0001 (Student’s t test)

To dig out a gene sharing the regulatory role of miR-7-5p and LPP-AS2, we used four online databases (TargetScan, miRWalk, miRTarBase and miRDB) to predict potential target genes of miR-7-5p (Fig. 6a), and discovered that 62 mRNAs might be potential targets of miR-7-5p including EGFR, as previously validated. Then, dual luciferase assays were performed to confirm the relationship between miR-7-5p and EGFR. The results indicated that compared with the EGFR-MUT group, the fluorescence intensity of cells co-transfected with EGFR-WT plasmids and miR-7-5p mimics markedly decreased. However, no significant differences were observed with the co-transfection of EGFR-MUT plasmids and miR-7-5p mimics (Fig. 6b). Moreover, the influence of miR-7-5p on the mRNA and protein levels of EGFR was measured. The results indicated that inhibited expression of miR-7-5p significantly enhanced the level of EGFR, whereas the level of EGFR was reduced by transfection of miR-7-5p mimics in U251 and SHG44 cells (Fig. 6c, d and Figure S9a). Correlation analysis showed a negative correlation between expression of miR-7-5p and EGFR in 21 glioblastoma tissues (Fig. 6e).

Previous studies have demonstrated that EGFR leads to receptor tyrosine kinase autophosphorylation and further activated downstream intracellular signaling cascades especially the phosphatidylinositol 3-kinase-AKT serine/threonine kinase 1 (PI3K-AKT) pathway, by binding with its cognate ligands [67, 68]. Accordingly, we further investigated the regulatory ability of the LPP-AS2/miR-7-5p axis to influence EGFR expression and activate the downstream PI3K/AKT/c-MYC pathway. LPP-AS2 overexpression/knockdown distinctly increased/reduced the mRNA and protein levels of EGFR, phospho-PI3K (p-PI3K), phospho-AKT (p-AKT) and c-MYC protein, results that were partially reversed by co-transfection with miR-7-5p mimics/inhibitors. Conversely, the total protein level of PI3K or AKT was almost unchanged (Fig. 6f-j and Figure S9c-g). Besides, knockdown of LPP-AS2 remarkably reduced the level of EGFR and its downstream effectors phospho-PI3K, phospho-AKT and c-MYC, whereas no significant changes in the total protein level of PI3K or AKT in U251 and SHG44 cells were observed (Fig. 6k, l and Figure S9b). In contrast, overexpression of LPP-AS2 obviously elevated the protein levels of EGFR, phospho-PI3K, phospho-AKT and c-MYC, though the level of PI3K or AKT was not obviously altered (Figure S9h-j). Taken together, these findings demonstrate that LPP-AS2 decoys for miR-7-5p to upregulate expression of EGFR and stimulate the PI3K/AKT/c-MYC signaling pathway.

**Fig. 7:**
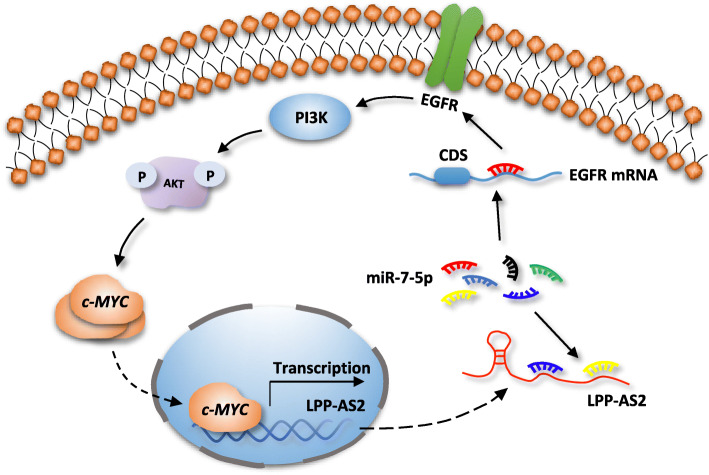
Schematic illustration of the proposed mechanism of LPP-AS2 in glioma

More importantly, we found that c-MYC could regulate expression of LPP-AS2, suggesting that LPP-AS2 is transcriptionally regulated by c-MYC, and functions as an oncogene in glioma via an miR-7-5p/EGFR/PI3K/AKT/c-MYC feedback loop (Fig. 7).

## Discussion

A growing number of studies have demonstrated that lncRNAs are important factors in human diseases and individual development. Abnormal expression of lncRNAs may lead to tumorigenesis and aggressive progression, rendering these molecules attractive therapeutic targets [61, 62]. For instance, a newly identified lncRNA called PTAR, the expression of which correlates significantly with tumor subtype, promotes the epithelial-mesenchymal transition (EMT) and metastasis in ovarian cancer via the miR-101-3p/ZEB1 pathway [61]. The proliferation and tumor formation abilities of gastric cancer cells are inhibited by knockdown of LINC01234, a potential molecular target of tumor therapy [62]. In addition, the lncRNA upregulated in colorectal cancer (CRC) liver metastasis (UICLM) is significantly increasing in cases of CRC with liver metastasis and leads to poor clinical survival [69]. However, the function and molecular mechanism of LPP-AS2 in tumors, including glioblastoma, have not been elucidated to date.

Glioblastoma, a devastating intracranial neoplasm with a high prevalence and poor clinical prognosis, is hardly achieved substantial progress through using surgical resection and chemoradiotherapy. Therefore, a novel potential effective therapeutic target is urgently needed. In this study, we used next-generation analysis and integrated GEO datasets to investigate the profiles of aberrantly expressed lncRNAs and mRNAs between glioblastoma and normal brain tissues. We then identified a novel lncRNA LPP-AS2, that shows distinctly upregulated expression in glioblastoma specimens and cell lines. This upregulated expression of LPP-AS2 was observed to be related to poor prognosis and adverse clinical survival among patients with glioblastoma. Gain-of-function and loss-of-function experiments demonstrated that LPP-AS2 promotes the occurrence and development of glioblastoma in vitro and in vivo. These data indicate that LPP-AS2 plays a carcinogenic role and deserves to be investigated with regard to its molecular mechanism in glioblastoma.

By comparing the differentially expressed genes in the results of high-throughput sequencing and constructing a co-expression network analysis, we identified a positive relationship between LPP-AS2 and EGFR, a well-known gene involved in cell proliferation and apoptosis via activation of the PI3K/AKT signaling pathway. Knockdown of LPP-AS2 decreased expression of EGFR in glioblastoma cells, whereas overexpression of LPP-AS2 elevated EGFR expression. Besides, the levels of phospho-PI3K, phospho-AKT, and c-MYC proteins were modulated by LPP-AS2. Furthermore, functional studies showed that overexpression/downregulation of EGFR could partially rescue the inhibition/promotion caused by silencing/elevating LPP-AS2 in glioblastoma cells. These findings indicated that LPP-AS2 regulated expression of EGFR and then activated the PI3K/AKT pathway to affect the biological behaviors of glioblastoma cells.

An increasing number of studies have demonstrated that the molecular mechanisms of lncRNAs greatly relied on their subcellular localization [70]. In addition, with the continuous understanding of tumor occurrence, a novel and extensive interaction network involving ceRNAs has been reported, in which lncRNAs could regulate expression of miRNAs by competitively binding to their target sites in protein-coding mRNA elements [71, 72]. For instance, lncRNA protein disulfide isomerase family A member 3 pseudogene 1 (PDIA3P1) affected chemotherapy sensitivity by acting as a microRNA sponge for miR-125a/b/miR-124 to elevate TRAF6 expression and augment NF-κB signaling pathway [73]. LncRNA LINC00336 suppressed ferroptosis and promoted the tumorigenesis of lung cancer through serving as a competitively endogenous sponge of miR-6852 to promote cystathionine-β-synthase expression [74]. In our research, we found that LPP-AS2 predominantly localized to the cytoplasm of glioblastoma cells, with an inverse relationship between LPP-AS2 and miR-7-5p by RT-qPCR, which indicated that LPP-AS2 may function through post-transcriptional regulation. Further evidence obtained by bioinformatics analysis, dual luciferase reporter assays, RNA immunoprecipitation, and RNA pulldown assays, confirmed that LPP-AS2 interacted with miR-7-5p by directly binding to its complementary sequence. Besides, emerging evidence demonstrated that miR-7-5p was downregulated in various cancers and acted as a tumor suppressor. For example, restoring expression of miR-7-5p suppressed tumorigenesis in colorectal cancer cells and reversed by SP1-induced lncRNA TINCR [65], and miR-7-5p was a potential biomarker in neuroendocrine neoplasms of the small intestine [66]. In this study, we found that miR-7-5p was distinctly downregulated in glioblastoma cells and that its overexpression repressed the biological behaviors of glioblastoma, which was consistent with the results of a recent report [35]. Moreover, the effects of silencing LPP-AS2 on proliferation and invasion were reversed by the miR-7-5p inhibitor, whereas transfecting miR-7-5p mimics into glioblastoma cells partially rescued the effects caused by overexpression of LPP-AS2. Taken together, our findings suggested that LPP-AS2 functioned as an oncogenic role in glioblastoma by promoting cell viability, which can be partially rescued by miR-7-5p mimics.

In general, miRNAs exert functions in cancer signaling pathways by depending on depression or degradation of target genes, thereby affecting cell fate and biological function [75]. In our study, we used four online databases to predict the potential target of miR-7-5p. Surprisingly, the target gene EGFR was observed in the intersection of four subsets, negatively correlating with miR-7-5p. The results of dual luciferase reporter assays indicated that EGFR directly bind to miR-7-5p, which was also in agreement with the previous reports concerning EGFR in glioblastoma and other diseases [35, 76]. Moreover, overexpression/downregulation of miR-7-5p repressed/promoted EGFR mRNA and protein expression. Given that both LPP-AS2 and EGFR interact with miR-7-5p, we hypothesized that LPP-AS2 indirectly regulates expression of EGFR by competitively binding to miR-7-5p, and we conducted RT-qPCR and western blotting to validate this hypothesis. The mRNA and protein levels of EGFR were downregulated by silencing LPP-AS2 and rescued by miR-7-5p inhibitor; overexpressing LPP-AS2 was also partially reversed by miR-7-5p mimics. Therefore, we demonstrated that LPP-AS2 regulates EGFR expression and activates the PI3K/AKT/c-MYC signaling pathway by competitively sponging miR-7-5p, thereby promoting glioblastoma cell viability.

As a transcriptional factor, c-MYC is regarded as a human proto-oncogene and leads to multiple features of cancer. Many studies have suggested that c-MYC transcriptional targets are involved in various biological processes, such as cell proliferation, apoptosis, and metabolism [77]. Interestingly, a recent study reported that LPP-AS2 is transcriptionally repressed in CRC cells with a high level of c-MYC, though the regulation may be indirect [27]. Nevertheless, it remains unclear whether c-MYC is able to transcriptionally regulate expression of LPP-AS2 in glioblastoma. Here, we show that knockdown of c-MYC significantly inhibits expression of LPP-AS2. ChIP was performed to prove that c-MYC specifically binds to the promoter of LPP-AS2 and elevates LPP-AS2 expression in glioblastoma. These results indicate the existence of a positive feedback loop between LPP-AS2 and c-MYC, thus affecting the biological behaviors of glioblastoma cells.

## Conclusion

Our study revealed that elevated LPP-AS2 expression was a common event in glioblastoma and that aberrant expression of LPP-AS2 was significantly associated with a poor prognosis in glioblastoma patients. LPP-AS2 functioned as an oncogenic lncRNA in glioblastoma both in vitro and in vivo. In addition, LPP-AS2 was involved in regulating EGFR expression by competitively sponging miR-7-5p and then stimulating the PI3K/AKT/c-MYC pathway; it was also transcriptionally regulated by c-MYC. Overall, our study provided new mechanistic insight into the pathogenesis of glioblastoma and suggested a potential novel therapeutic target for glioma patients.

## Supplementary information

**Additional file 1: Figure S1.** Identification of differentially expressed lncRNAs and mRNAs in high-throughput RNA sequencing. **a** Heatmap of reported lncRNAs and mRNAs in high-throughput RNA sequencing. **b** Hierarchical cluster analysis of differentially expressed mRNAs in three glioblastoma and normal samples. **c** Volcano plot of differentially expressed mRNAs in three glioblastoma and normal samples. **Figure S2.** LPP-AS2 is upregulate in glioblastoma and regulated by c-MYC. **a** Four dysregulated lncRNAs obtained from RNA-seq were validated in 6 normal brain tissues and 15 GBM tissues by RT-qPCR. **b** Six dysregulated mRNAs obtained from RNA-seq were validated in 6 normal brain tissues and 15 GBM tissues by RT-qPCR. **c** Co-expression network of 35 mRNAs in the neuron apoptotic process and the top 50 lncRNAs. **d** Relative expression level of LPP-AS2 in TCGA (207 normal brain tissues and 163 glioma tissues). **e** Relative LPP-AS2 expression in glioma tissues (*n* = 75) and normal brain tissues (*n* = 13) of the GEO databases (GSE50161 and GSE33331). **f** Relative expression of LPP-AS2 in U251 and SHG44 cells treated with siRNAs of c-MYC. Error bars, s.e.m. from three independent experiments. *P < 0.05; **P < 0.01; ***P < 0.001; ****P < 0.0001 (Student’s t test). **Figure S3.** LPP-AS2 promotes glioblastoma progression in vitro. **a** Transwell assays in U251 and SHG44 cells transfected with siRNAs of LPP-AS2 and treated with Ara-C. Representative staining images are presented. **b** Transwell assays in U251 and SHG44 cells transfected with overexpression of LPP-AS2 and treated with Ara-C. Representative staining images are presented. **c** Wound healing assays in U251 and SHG44 cells transfected with siRNAs/plasmids of LPP-AS2 and treated with Ara-C. Data represent the mean ± s.e.m. of three independent experiments. **P < 0.01; ***P < 0.001; ****P < 0.0001 (Student’s t test). **Figure S4.** Regulation relationship between LPP-AS2 and EGFR. **a** Relative expression of LPP in U251 and SHG44 cells treated with siRNAs of LPP-AS2. **b** Relative expression of LPP in U251 and SHG44 cells with overexpression of LPP-AS2. **c** Relative expression of EGFR in seven glioma cell lines and HEB by RT-qPCR. **d** Overexpression of LPP-AS2 significantly increased EGFR expression in U251 and SHG44 cells. **e** Protein level of EGFR in U251 and SHG44 cells with overexpression of LPP-AS2. Error bars, s.e.m. from three independent experiments. ns (no significance); *P < 0.05; **P < 0.01; ***P < 0.001; ****P < 0.0001; (Student’s t test). **Figure S5.** LPP-AS2 requires EGFR to facilitate tumor progression. **a** Relative expression of EGFR in U251 and SHG44 cells transfected with siRNAs of EGFR alone or co-transfection of pEGFP-C1/LPP-AS2. **b** CCK8 assays in U251 and SHG44 cell treated with siRNAs of EGFR alone or co-transfection of pEGFP-C1/LPP-AS2. **c** Transwell assays in U251 and SHG44 cells treated with siRNAs of EGFR alone or co-transfection of pEGFP-C1/LPP-AS2. **d** Migration assays in U251 and SHG44 cells treated with siRNAs of EGFR alone or co-transfection of pEGFP-C1/LPP-AS2. **e** Colony formation assays in U251 and SHG44 cells treated with siRNAs of EGFR alone or co-transfection of pEGFP-C1/LPP-AS2. **f** Flow cytometry assays in U251 and SHG44 cells treated with siRNAs of EGFR alone or co-transfection of pEGFP-C1/LPP-AS2. Error bars, s.e.m. from three independent experiments. *P < 0.05; **P < 0.01; ***P < 0.001; ****P < 0.0001; ^#^*P* < 0.05; ^##^*P* < 0.01; ^###^*P* < 0.001 (Student’s t test). **Figure S6.** Characterization of miR-7-5p in glioma. **a** Relative expression of miR-7-5p in 17 normal brain tissues and 17 glioblastoma tissues. **b** Relative expression of miR-7-5p in seven glioma cell lines and HEB by RT-qPCR. **c** Relative expression of miR-7-5p in U251 and SHG44 cells treated with Overexpression of LPP-AS2. **d** Knockdown or overexpression of miR-7-5p in U251 and SHG44 cells have no significantly effect on LPP-AS2 expression. e Relative expression of miR-7-5p in U251 and SHG44 cells treated with miR-7-5p mimics alone or co-transfection of pEGFP-C1/LPP-AS2. Data represent the mean ± s.e.m. of three independent experiments. ns (no significance); **P < 0.01; ***P < 0.001; ****P < 0.0001; ^##^*P* < 0.01; ^###^*P* < 0.001; ^####^*P* < 0.0001 (Student’s t test). **Figure S7.** Functions of miR-7-5p in glioma. **a** CCK8 assays in U251 and SHG44 cell transfected with miR-7-5p mimics or miR-7-5p inhibitor. **b** Colony formation assays in U251 and SHG44 cells transfected with miR-7-5p mimics or miR-7-5p inhibitor. **c** Transwell assays in U251 and SHG44 cells transfected with miR-7-5p mimics or miR-7-5p inhibitor. **d** Migration assays in U251 and SHG44 cells transfected with miR-7-5p mimics or miR-7-5p inhibitor. **e** Flow cytometry assays in U251 and SHG44 cells transfected with miR-7-5p mimics or miR-7-5p inhibitor. Error bars, s.e.m. from three independent experiments. *P < 0.05; **P < 0.01; ***P < 0.001; ****P < 0.0001 (Student’s t test). **Figure S8.** LPP-AS2 decoys miR-7-5p to regulate glioma progression. **a** CCK8 assays in U251 and SHG44 cell transfected with miR-7-5p mimics alone or co-transfection of pEGFP-C1/LPP-AS2. **b** Migration assays in U251 and SHG44 cells transfected with miR-7-5p mimics alone or co-transfection of pEGFP-C1/LPP-AS2. Migration assays in U251 and SHG44 cells transfected with miR-7-5p inhibitor alone or co-transfection of siRNAs of LPP-AS2. **c** Transwell assays in U251 and SHG44 cells transfected with miR-7-5p mimics alone or co-transfection of pEGFP-C1/LPP-AS2. Representative staining images are presented. **d** Colony formation assays in U251 and SHG44 cells transfected with miR-7-5p mimics alone or co-transfection of pEGFP-C1/LPP-AS2. **e** Flow cytometry assays in U251 and SHG44 cells transfected with miR-7-5p mimics alone or co-transfection of pEGFP-C1/LPP-AS2. Error bars, s.e.m. from three independent experiments. *P < 0.05; **P < 0.01; ***P < 0.001; ****P < 0.0001; ^#^*P* < 0.05; ^##^*P* < 0.01; ^###^*P* < 0.001 (Student’s t test). **Figure S9.** LPP-AS2 functions as an oncogene via miR-7-5p/EGFR/PI3K/AKT/c-MYC axis. **a** Relative protein levels of EGFR in U251 and SHG44 cells treated with miR-7-5p mimics or miR-7-5p inhibitor. **b** Relative protein levels of EGFR in U251 and SHG44 cells treated with pEGFP-C1/EGFR alone or co-transfection of si-LPP-AS2–1. **c** Western blotting analysis of EGFR, PI3K, AKT, p-PI3K, p-AKT, c-MYC in U251 and SHG44 cells treated with miR-7-5p mimics alone or co-transfection with pEGFP-C1/LPP-AS2. **d** Histogram of EGFR protein levels in U251 and SHG44 cells treated with miR-7-5p mimics alone or co-transfection with pEGFP-C1/LPP-AS2. **e** Relative expression of EGFR in U251 and SHG44 cells treated with miR-7-5p mimics alone or co-transfection with pEGFP-C1/LPP-AS2 were validated by RT-qPCR. **f, g** Ratio of p-PI3K/PI3K and p-AKT/AKT in U251 and SHG44 cells. **h** Western blotting analysis of EGFR, PI3K, p-PI3K, AKT, p-AKT and c-MYC in U251 and SHG44 cells treated with siRNAs of EGFR alone or co-transfection with pEGFP-C1/LPP-AS2. **i** Histogram of EGFR protein levels in U251 and SHG44 cells treated with siRNAs of EGFR alone or co-transfection with pEGFP-C1/LPP-AS2. **j** Ratio of p-PI3K/PI3K and p-AKT/AKT in U251 and SHG44 cells treated with siRNAs of EGFR alone or co-transfection with pEGFP-C1/LPP-AS2. Data represent the mean ± s.e.m. of three independent experiments. *P < 0.05; **P < 0.01; ***P < 0.001; ****P < 0.0001; ^#^*P* < 0.05; ^##^*P* < 0.01; ^###^*P* < 0.001; ^####^*P* < 0.0001 (Student’s t test).

**Additional file 2: Table S1.** Oligos used in the research.

## Data Availability

All data in this study are available upon request.

## Abbreviations

lncRNA: Long non-coding RNA
LPP-AS2: LPP antisense RNA 2
ceRNA: Competing endogenous RNA
EGFR: Epidermal growth factor receptor
RT-qPCR: Real-time quantitative PCR
IHC: Immunohistochemistry
RIP: RNA immunoprecipitation
GEO: Gene Expression Omnibus
ChIP: Chromatin immunoprecipitation
TCGA: The Cancer Genome Atlas
