# Supplementary material for: Long non-coding RNA LPP-AS2 promotes glioma tumorigenesis via miR-7-5p/EGFR/PI3K/AKT/c-MYC feedback loop
Source: J Exp Clin Cancer Res. 2020 Sep 22;39:196. doi: 10.1186/s13046-020-01695-8 (PMC7510091; doi:10.1186/s13046-020-01695-8)

Supplementary figure 1

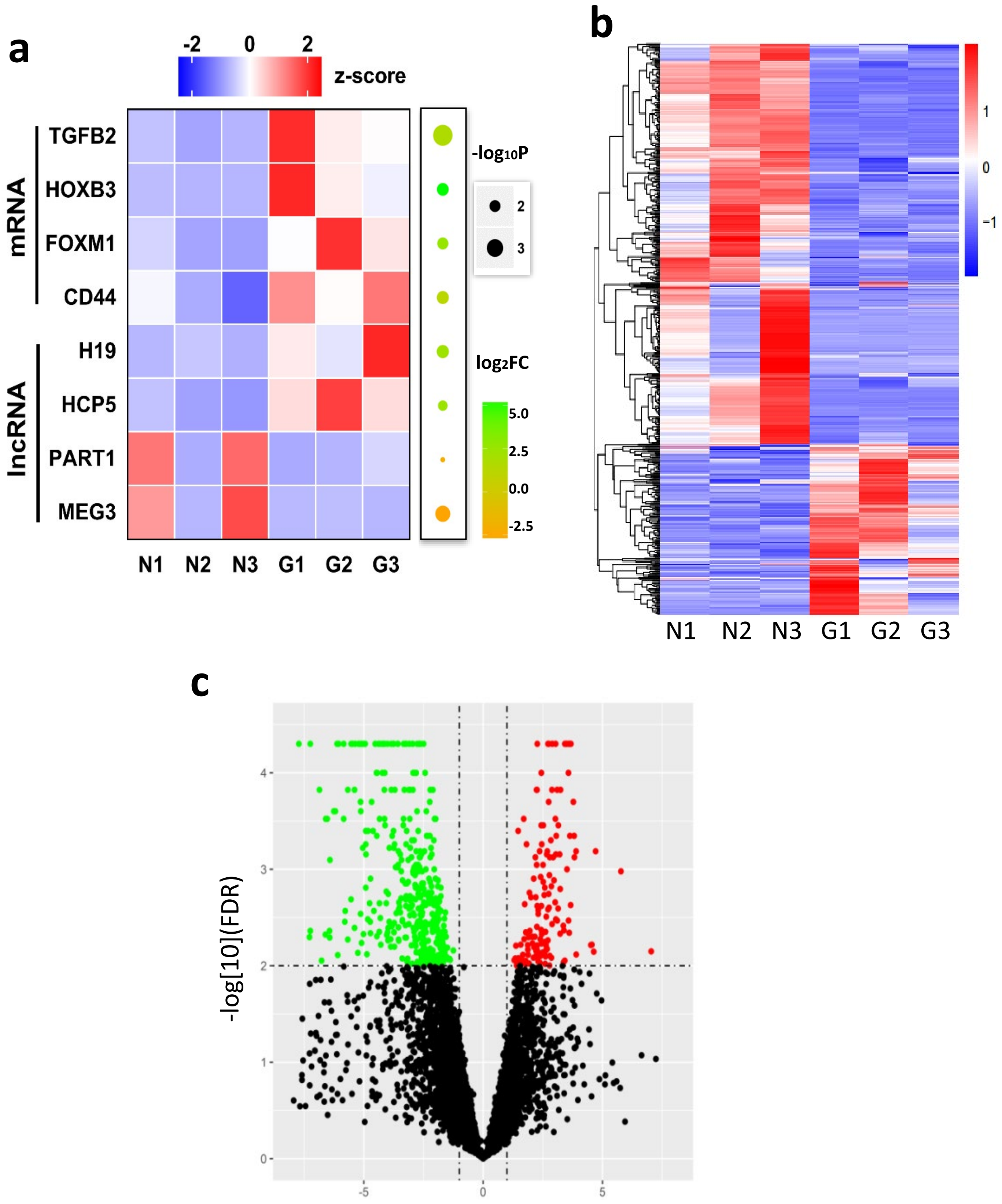

Supplementary figure 2

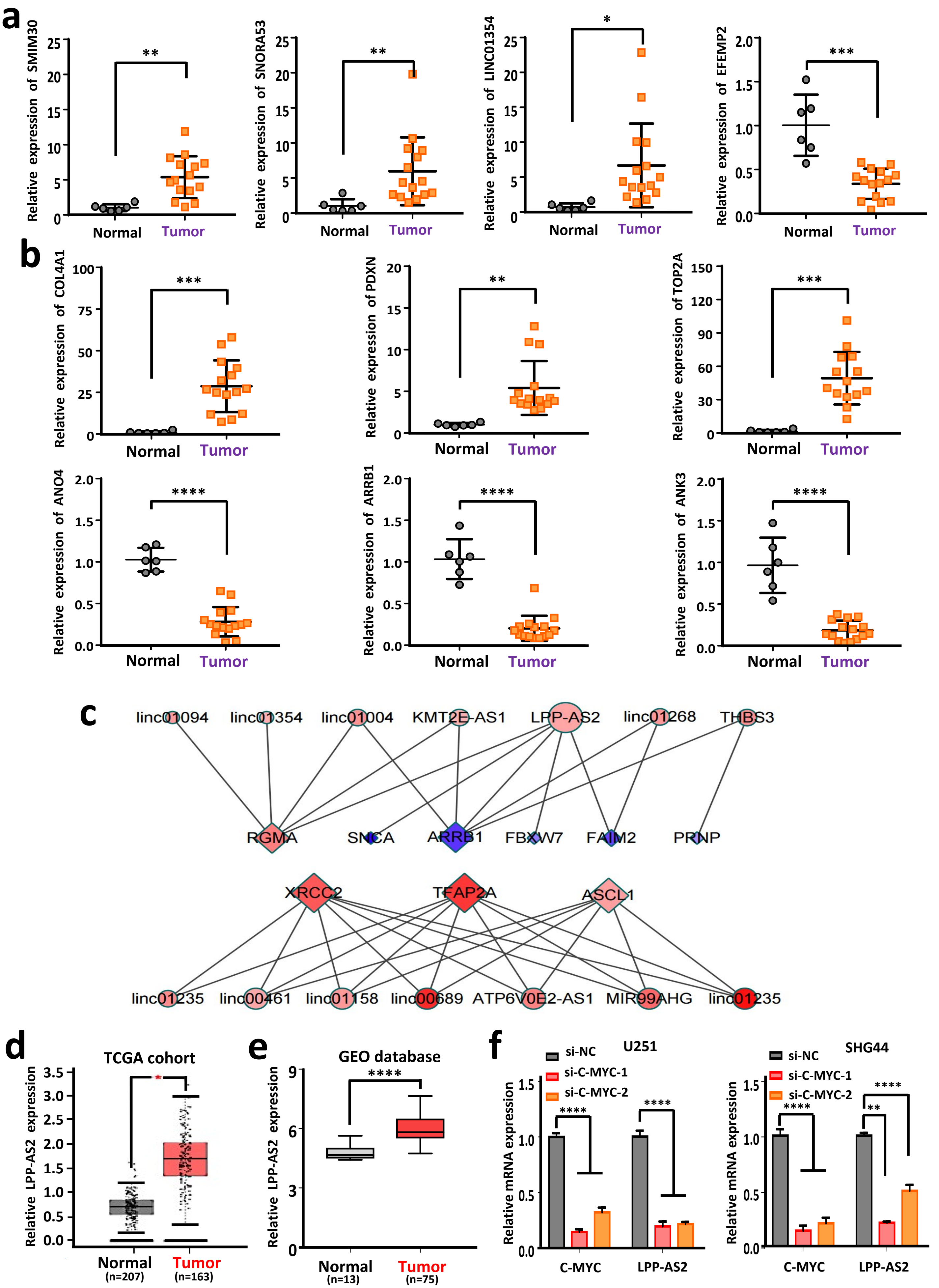

Supplementary figure 3

a

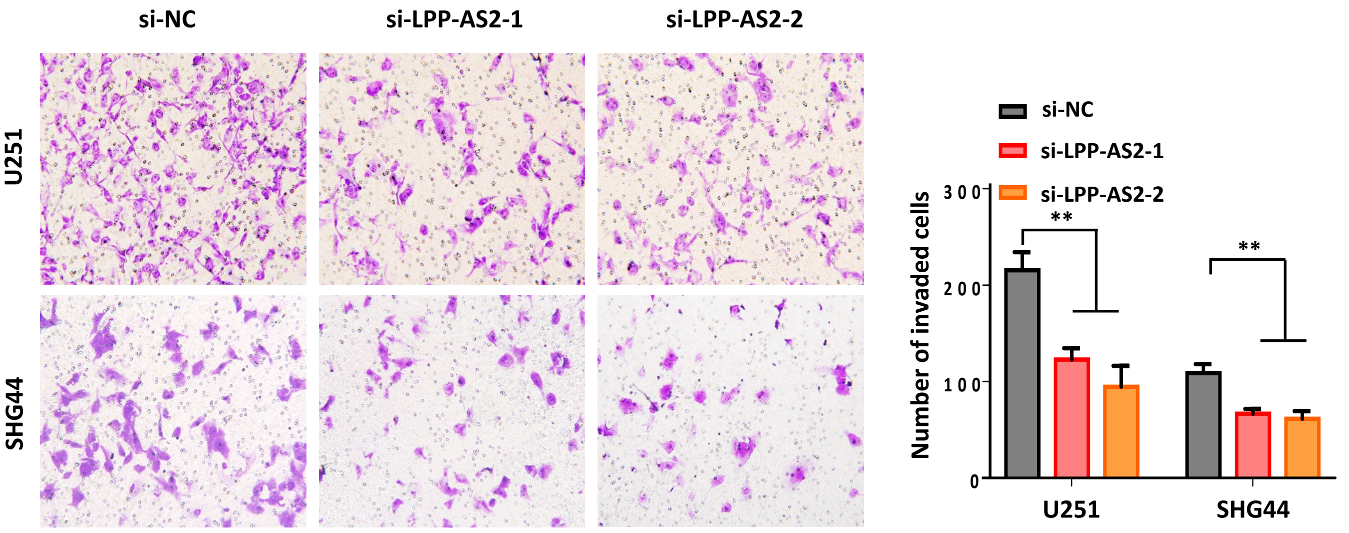

b

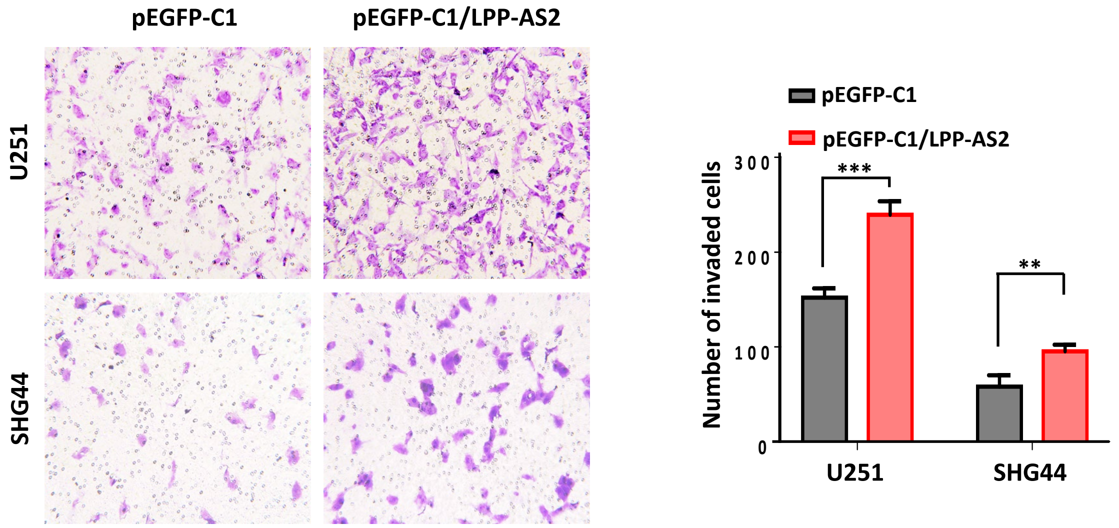

c

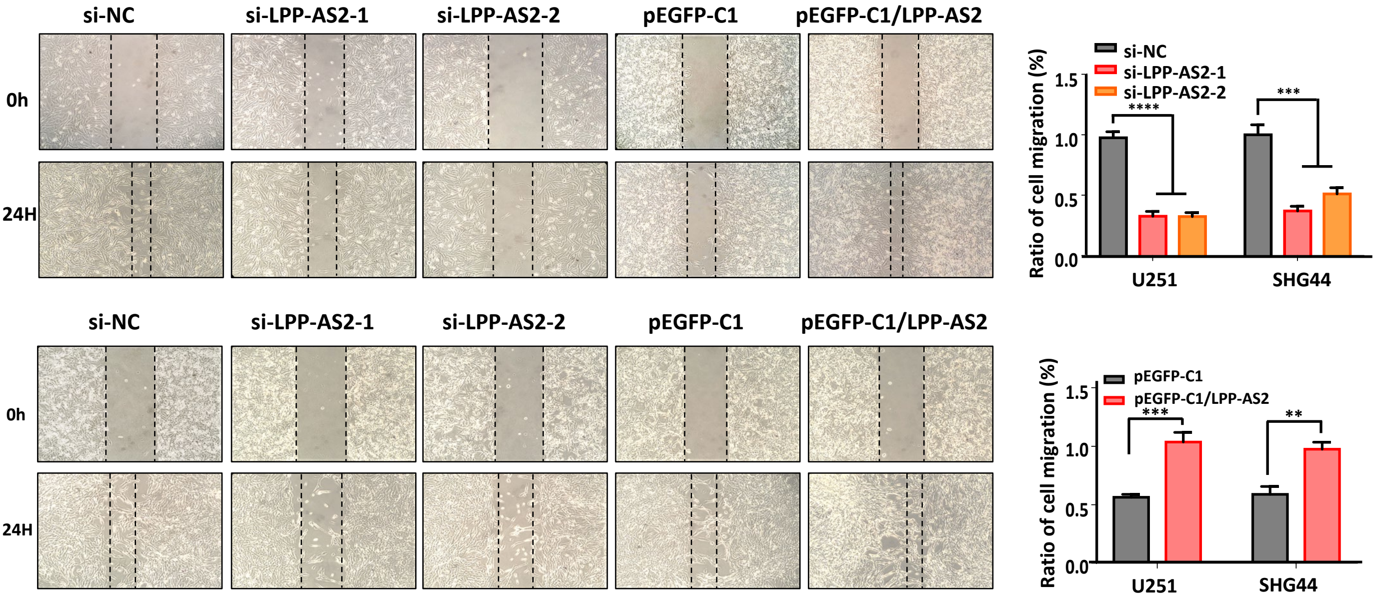

Supplementary figure 4

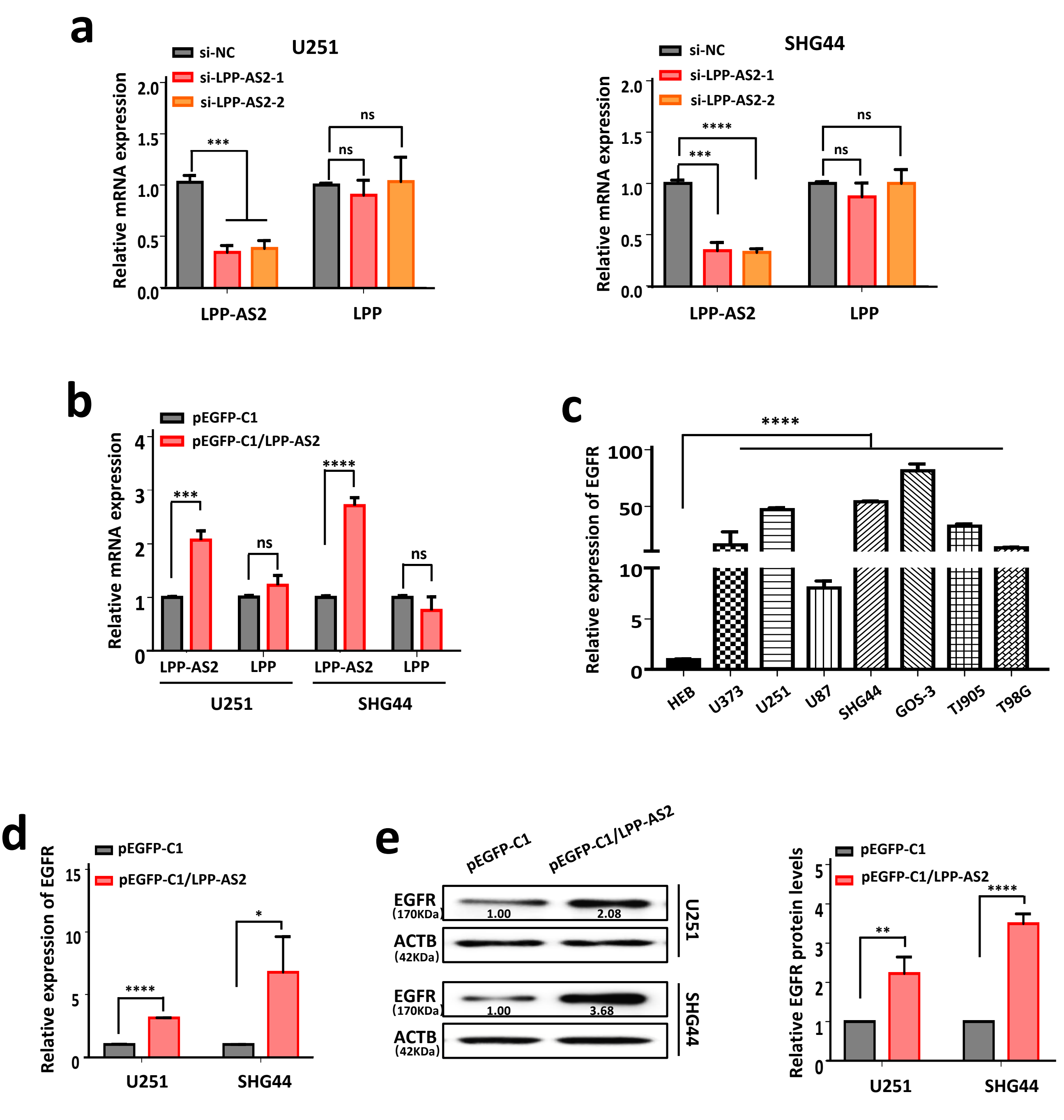

Supplementary figure 5

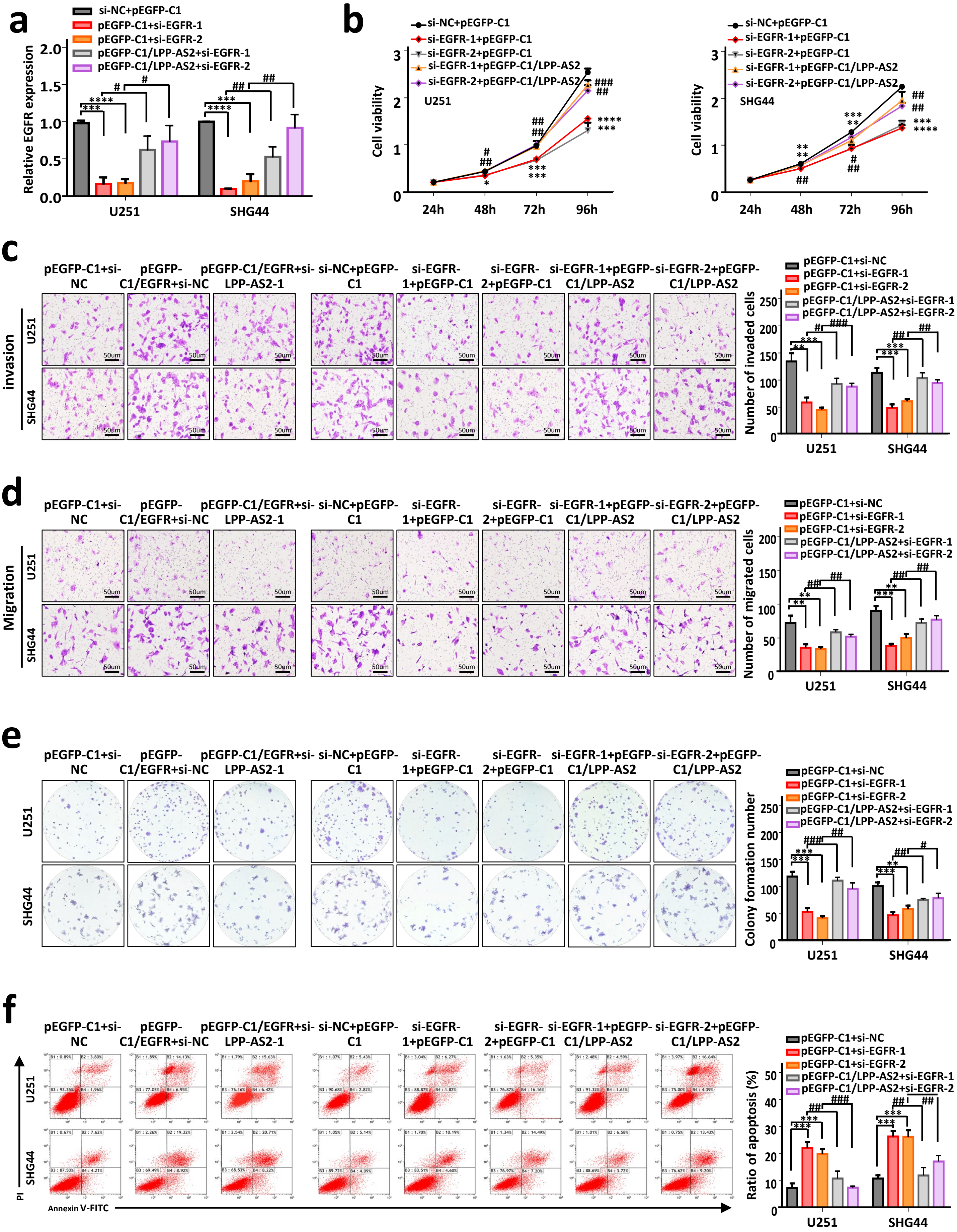

Supplementary figure 6

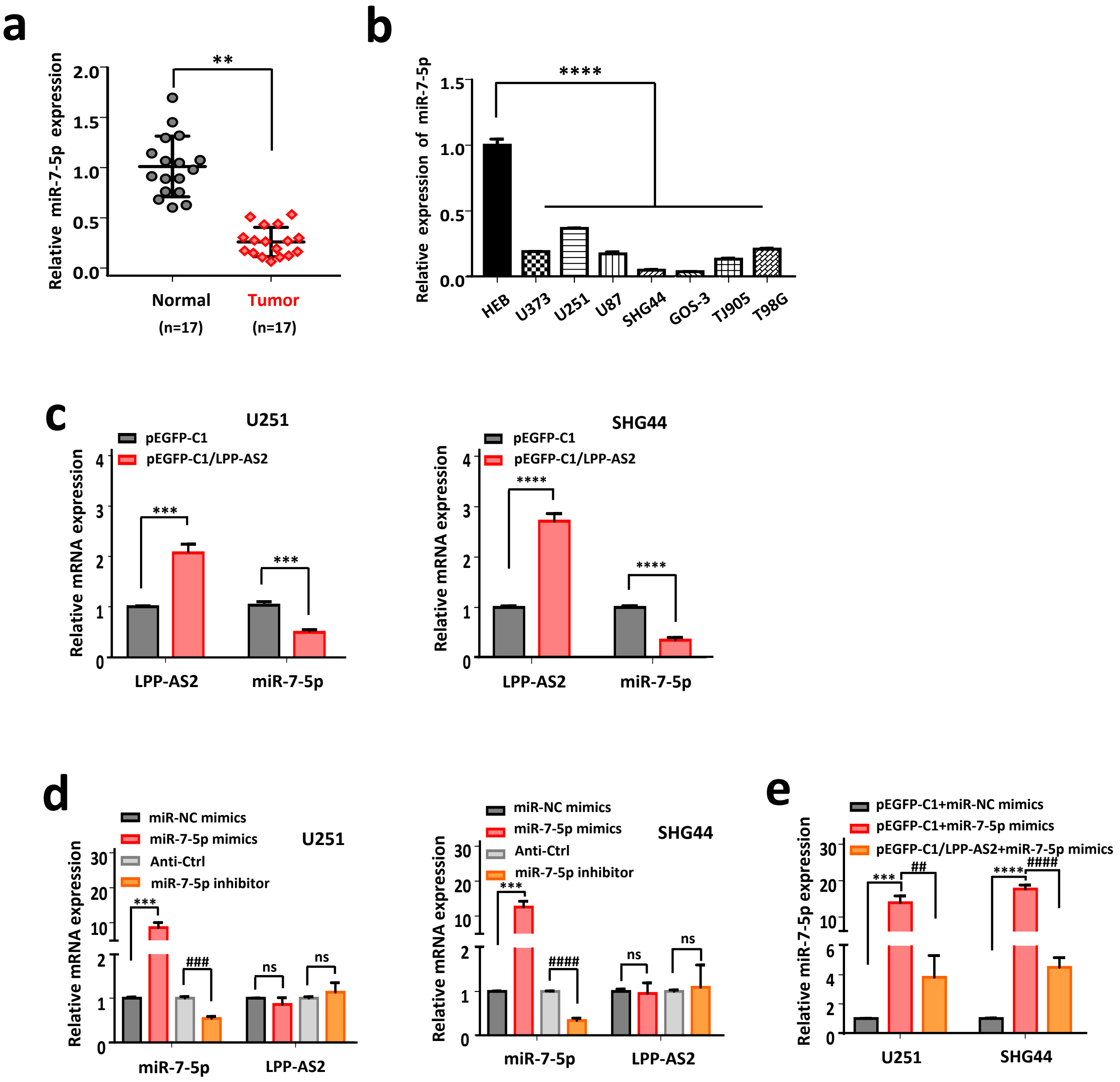

Supplementary figure 7

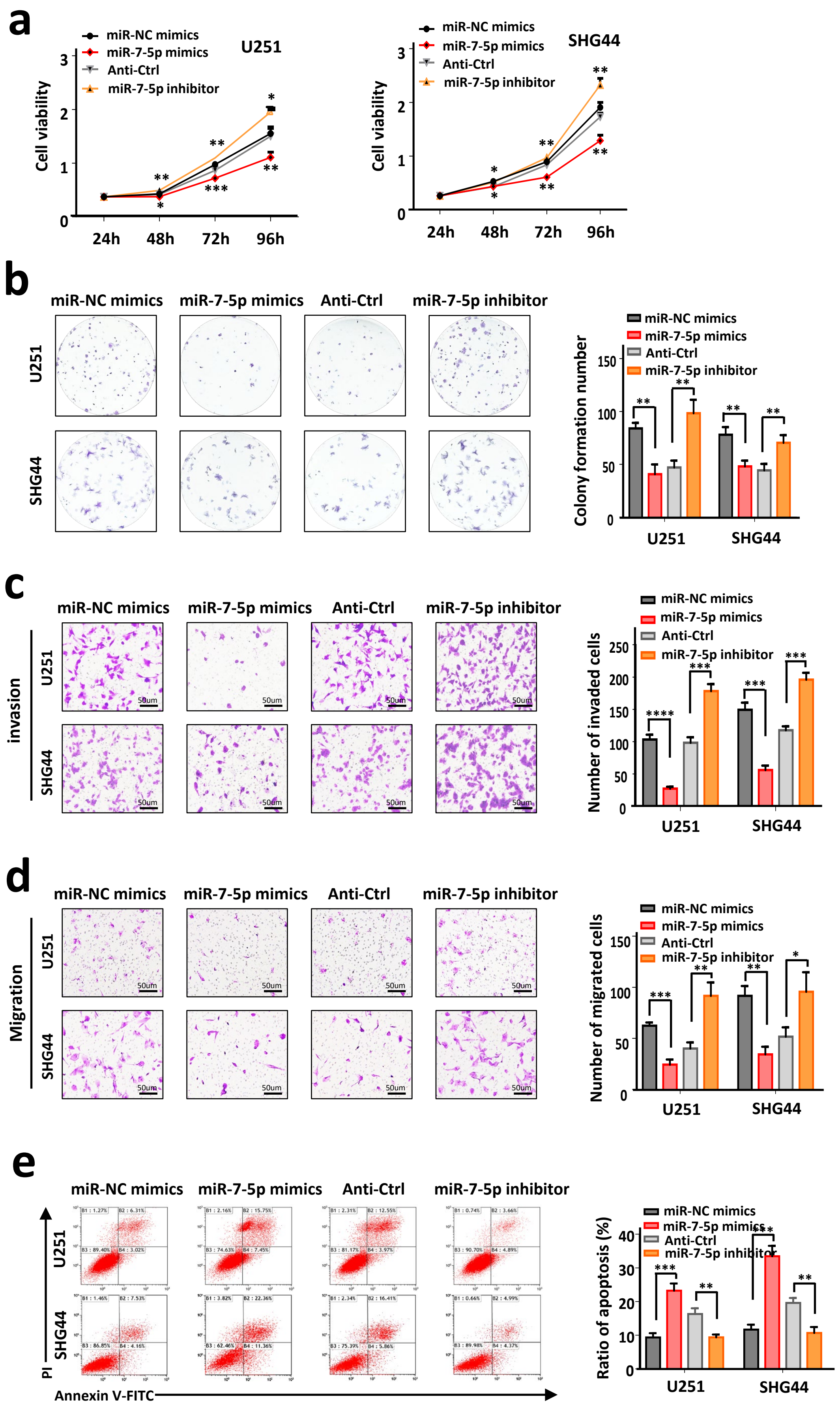

Supplementary figure 8

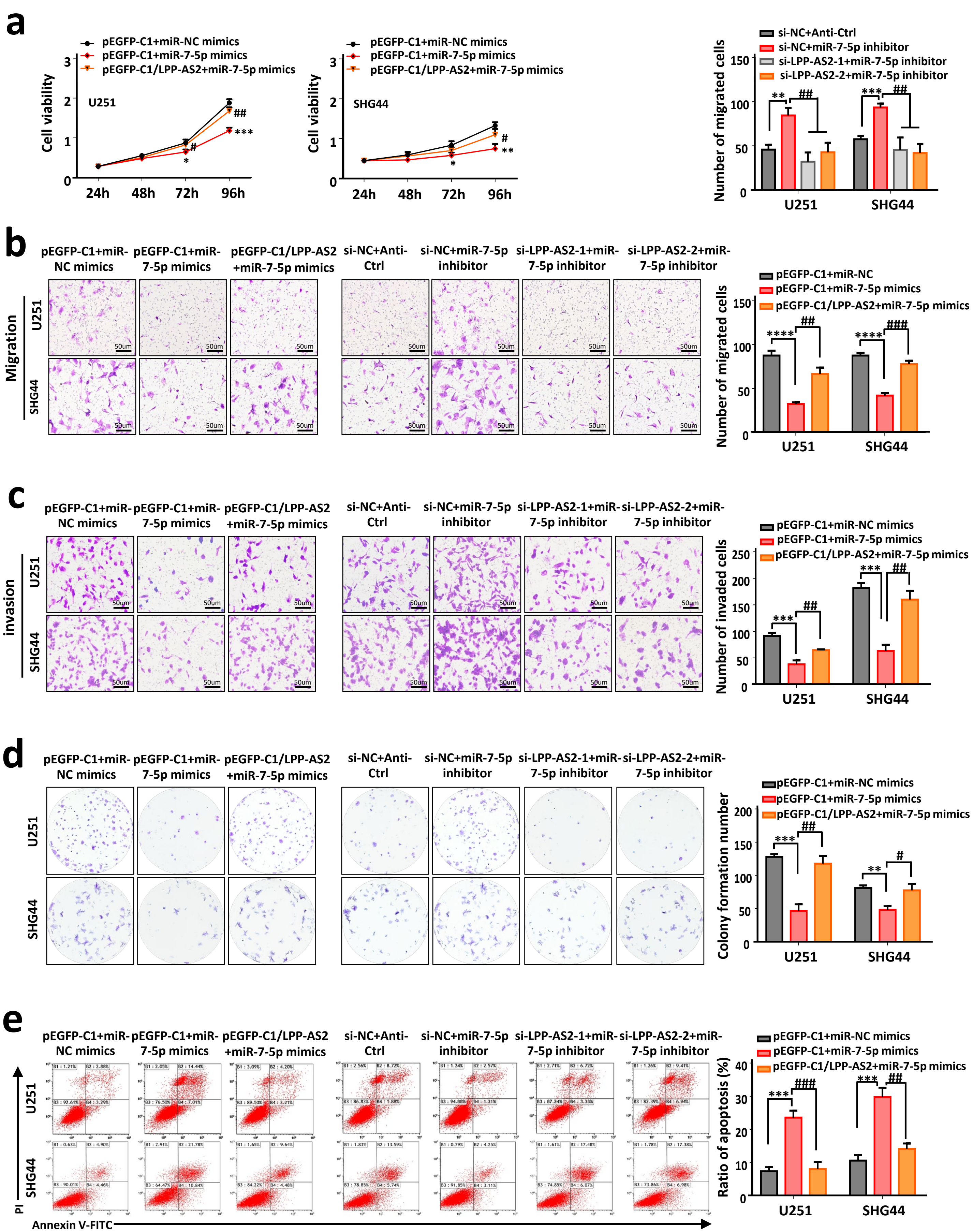

Supplementary figure 9

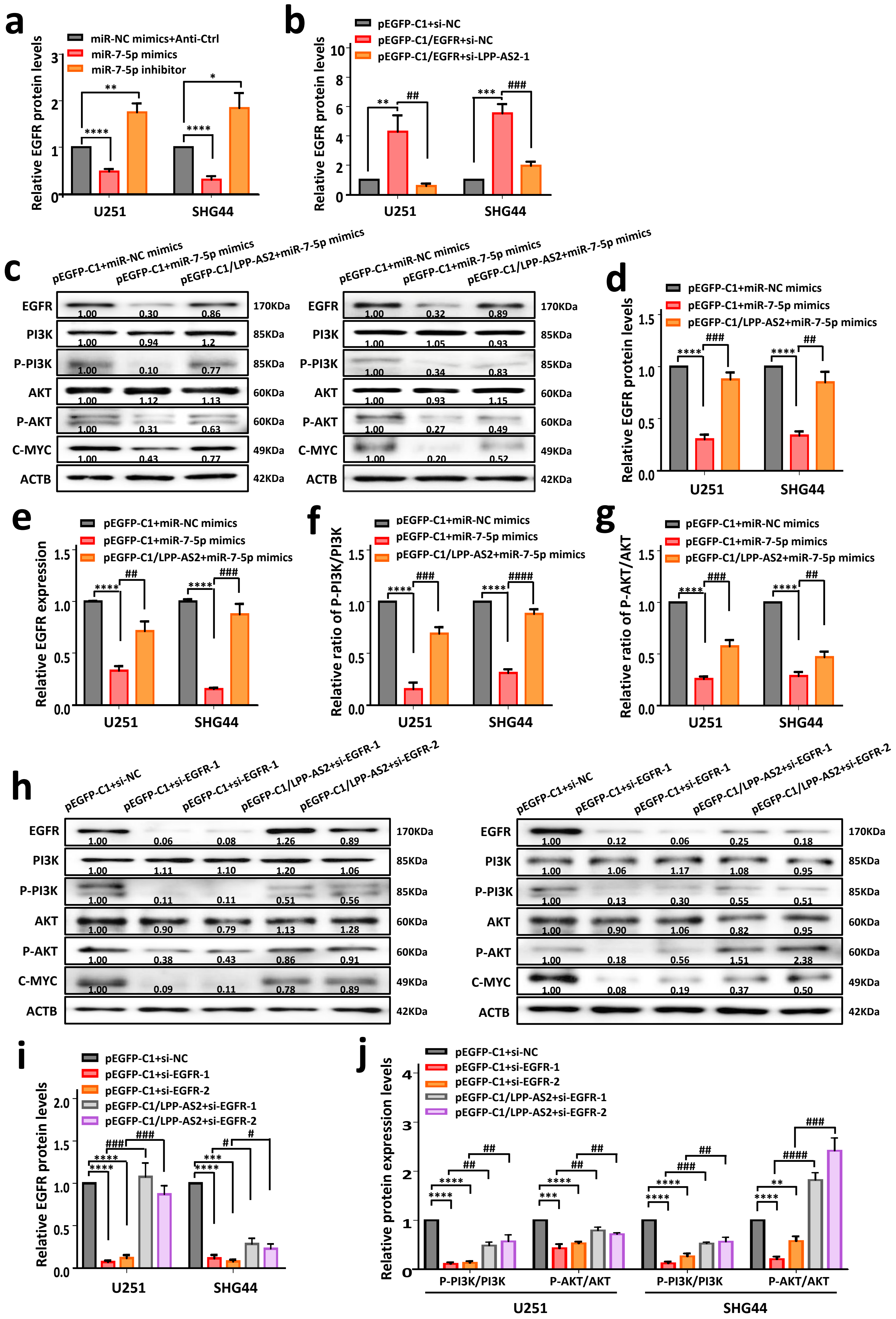

Supplement: Supplementary file 1 — Additional file 1: Figure S1. Identification of differentially expressed lncRNAs and mRNAs in high-throughput RNA sequencing. a Heatmap of reported lncRNAs and mRNAs in high-throughput RNA sequencing. b Hierarchical cluster analysis of differentially expressed mRNAs in three glioblastoma and normal samples. c Volcano plot of differentially expressed mRNAs in three glioblastoma and normal samples. Figure S2. LPP-AS2 is upregulate in glioblastoma and regulated by c-MYC. a Four dysregulated lncRNAs obtained from RNA-seq were validated in 6 normal brain tissues and 15 GBM tissues by RT-qPCR. b Six dysregulated mRNAs obtained from RNA-seq were validated in 6 normal brain tissues and 15 GBM tissues by RT-qPCR. c Co-expression network of 35 mRNAs in the neuron apoptotic process and the top 50 lncRNAs. d Relative expression level of LPP-AS2 in TCGA (207 normal brain tissues and 163 glioma tissues). e Relative LPP-AS2 expression in glioma tissues (n = 75) and normal brain tissues (n = 13) of the GEO databases (GSE50161 and GSE33331). f Relative expression of LPP-AS2 in U251 and SHG44 cells treated with siRNAs of c-MYC. Error bars, s.e.m. from three independent experiments. *P < 0.05; **P < 0.01; ***P < 0.001; ****P < 0.0001 (Student’s t test). Figure S3. LPP-AS2 promotes glioblastoma progression in vitro. a Transwell assays in U251 and SHG44 cells transfected with siRNAs of LPP-AS2 and treated with Ara-C. Representative staining images are presented. b Transwell assays in U251 and SHG44 cells transfected with overexpression of LPP-AS2 and treated with Ara-C. Representative staining images are presented. c Wound healing assays in U251 and SHG44 cells transfected with siRNAs/plasmids of LPP-AS2 and treated with Ara-C. Data represent the mean ± s.e.m. of three independent experiments. **P < 0.01; ***P < 0.001; ****P < 0.0001 (Student’s t test). Figure S4. Regulation relationship between LPP-AS2 and EGFR. a Relative expression of LPP in U251 and SHG44 cells treated with siRNAs o [file 13046_2020_1695_MOESM1_ESM.pdf]
