# Supplementary material for: Long non-coding RNA LPP-AS2 promotes glioma tumorigenesis via miR-7-5p/EGFR/PI3K/AKT/c-MYC feedback loop
Source: J Exp Clin Cancer Res. 2020 Sep 22;39:196. doi: 10.1186/s13046-020-01695-8 (PMC7510091; doi:10.1186/s13046-020-01695-8)
Supplement: Supplementary file 2 — Additional file 2: Table S1. Oligos used in the research. [file 13046_2020_1695_MOESM2_ESM.docx]

**Table S1**: Oligos used in the research.

| GAPDH-F | CTTCATTGACCTCAACTACATGG | For human GAPDH RT-qPCR |
| --- | --- | --- |
| GAPDH-R | CTCGCTCCTSGGAAGATGGTGAT |  |
| LPP-AS2-F | CACTGAGCTTGAGTGGAAGA | For human LPP-AS2 RT-qPCR |
| LPP-AS2-R | TTCCTCACGTGCCAAGCGGA |  |
| Hsa-miR-7-5P-F | CACGCATGGAAGACTAGT | For human miR-7-5p RT-qPCR |
| Hsa-miR-7-5P-R | CCAGTGCAGGGTCCGAGGTA |  |
| EGFR-F | CAGCGCTACCTTGTCATTCA | For human EGFR RT-qPCR |
| EGFR-R | TGCACTCAGAGAGCTCAGGA |  |
| Q-U6-F | CGCTTCGGCAGCACATATAC | For U6 qPCR in human |
| Q-U6-R | TTCACGAATTTGCGTGTCAT |  |
| Q-c-MYC-F | AGCTGCTTAGACGCTGGATTTT | For c-MYC qPCR in human |
| Q-c-MYC-R | TCGAGGTCATAGTTCCTGTTGG |  |
| pre-GAPDH-F | CTGCTCACATATTCTGGAG | Primes for ChIP assays |
| pre-GAPDH-R | GTTAAAAGCAGCCCTGGTG |  |
| ChIP-LPP-AS2-1-F | TCCTGAGCTCAGGCAATCCG |  |
| ChIP-LPP-AS2-1-R | TGCTTGCACAGTTCCTGCCA |  |
| ChIP-LPP-AS2-2-F | TAAGTCCGGAGCCAACTATT |  |
| ChIP-LPP-AS2-2-R | ATCACAGTTGTCACAGCGGA |  |
| LPP-AS2 oligo1 | CATCTAACCTGCTTCAAGGCTCAGGGCTAA | Human LPP-AS2 antisense oligo with 5’biotin labeled |
| LPP-AS2 oligo2 | AATGACAGGCTTGTCTGTATTCAAACCAGT |  |
| LPP-AS2 oligo3 | GCTCTGTCCTATCTAACTCACCTGAAGATG |  |
| LPP-AS2 oligo4 | CTAACTCACCTGAAGATGCAATAGCCGGGT |  |
| LPP-AS2 oligo5 | TAGTCTTACCCCTGAAATGTCTGAATTCAA |  |
| LPP-AS2 oligo6 | TAGTACTCAGAATGTGAGTACGCTGAAATG |  |
| LPP-AS2 oligo7 | ATTAGTAGCATTTCTGGATCCGGCACAGTA |  |
| Scramble | TTCTCCGAACGTGTCACGTTCGAACGTGTC | Control oligo with  5’biotin labeled |
| MiR-7-5p scramble | CGAACGTGTCACGTTCGAACGTGT | Control oligo with  5’biotin labeled |
| MiR-7-5p oligo | AACAACAAAATCACTAGTCTTCCA | Human miR-7-5p antisense oligo with 5’biotin labeled |
| si-LPP-AS2-NC | UUCUCCGAACGUGUCACGU  ACGUGACACGUUCGGAGAA | Negative control |
| si-LPP-AS2-1 | UCUAACUCACCUGAAGAUGCA  UGCAUCUUCAGGUGAGUUAGA | siRNAs of LPP-AS2 |
| si-LPP-AS2-2 | AUACUAAACAGUAGUACUCAG  CUGAGUACUACUGUUUAGUAU |  |
| si-EGFR-1 | GCAAAGUGUGUAACGGAAUAGGUAU  AUACCUAUUCCGUUACACACUUUGC | siRNAs of EGFR |
| si-EGFR-2 | GGACUUCUUUCCCAAGGAA  UUCCUUGGGAAAGAAGUCC |  |
| si-c-MYC-1 | AAUGUUGGUGAAGCUAACGUU  AACGUUAGCUUCACCAACAUU | siRNAs of c-MYC |
| si-c-MYC-2 | UGUGUUCGCCUCUUGACAU  AUGUCAAGAGGCGAACACA |  |
